# Receptor‐targeting nanomaterials alleviate binge drinking‐induced neurodegeneration as artificial neurotrophins

**DOI:** 10.1002/EXP.20210004

**Published:** 2021-09-01

**Authors:** Jingyu Yang*, Lirong Wang*, Liwen Huang, Xiaohang Che, Zhen Zhang, Chunxiao Wang, Lihuan Bai, Ping Liu, Yanan Zhao, Xiaomei Hu, Bingyang Shi, Yuequan Shen, Xing‐Jie Liang, Chunfu Wu, Xue Xue

**Affiliations:** ^1^ Department of Pharmacology Shenyang Pharmaceutical University Shenyang P. R. China; ^2^ CAS Key Laboratory of Standardization and Measurement for Nanotechnology National Center for Nanoscience and Technology of China Beijing P. R. China; ^3^ State Key Laboratory of Medicinal Chemical Biology College of Pharmacy Nankai University Tianjin P. R. China; ^4^ CAS Center for Excellence in Nanoscience CAS Key Laboratory for Biomedical Effects of Nanomaterials and Nanosafety National Center for Nanoscience and Technology of China Beijing P. R. China; ^5^ International Joint Center for Biomedical Innovation School of Life Sciences Henan University Kaifeng Henan P. R. China

**Keywords:** artificial neurotrophins, binge drinking, carbon nanotubes

## Abstract

The distinguished properties of nanomaterials promote us to explore whether their intrinsic activities would be beneficial to disease treatment. Furthermore, understanding the molecular mechanism is thereby crucial for biomedical applications. Here, we investigate the therapeutic effects of single‐walled carbon nanotubes (SWNTs) in a rat model of binge alcohol‐induced neurodegeneration. With selection from four types of SWNT structures, bundled SWNTs (bSWNTs) facilitated the recovery of learning and memory via enhancing neuroprotection and neuroregeneration. We screened the potential target for bSWNTs, and found that bSWNTs have the abilities to directly interact with neurotrophic receptors, especially tropomyosin‐related kinase B (TrkB). Moreover, similar to the actions of endogenous neurotrophins, bSWNTs could trigger the dimerization and phosphorylation of TrkB, while these conformational changes resulted in activating their downstream signals involved in neuroprotection and neuroregeneration. With relatively clear mechanisms, these “artificial neurotrophins” provide a proof‐of‐concept example as an efficiently therapeutic strategy for the treatment of neurodegenerative diseases.

## Graphical abstract

Bundled single‐walled carbon nanotubes, as artificial neurotrophins, mediate dimerization and phosphorylation of tropomyosin‐related kinase B, and stimulate downstream signaling pathways for neuronal survival and differentiation. These therapeutic effects alleviate EtOH‐induced neurodegeneration in a rat model.

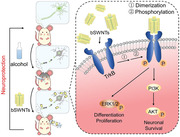

## 1 INTRODUCTION

Alcohol bingeing has brought disastrous health and social consequences to human beings, leading to the third risky for morbidity and disability.^[^ ^1^ ^]^ Binge drinking impairs brain development, and subsequently engages stress, anxiety, and depression, which can be transiently alleviated through further alcohol use, resulting in a binge‐drinking cycle.^[^ ^2^ ^]^ Previous studies also reported binge‐drinking‐induced brain injury share representative pathologies with Alzheimer's disease (AD), Parkinson's disease (PD), amyotrophic lateral sclerosis (ALS), classic neurodegenerative diseases (NDDs), and so forth. Therefore, we choose a rat model of 4‐day binge alcohol‐induced neurodegeneration and try to search new solution for neurodegenerative therapy.

Although some neuroprotective agents have exerted therapeutic effects on reducing neuronal damage, these strategies still lack clinical efficacy. Recently, increasing pre‐clinical research noted neurotrophic supplement is an important strategy to alleviate neurodegenerative deficiency of binge drinking and to help stimulate neural regeneration for therapy.^[^ ^3^, ^4^ ^]^ Neurotrophins, such as brain‐derived neurotrophic factor (BDNF), and their high affinity tropomyosin‐related kinase (Trk) receptors are actively distributed throughout the brain and are involved in regulating many aspects of neuronal development and function, such as neuronal survival, differentiation, outgrowth, and synaptic plasticity.^[^ ^5^ ^]^ Unfortunately, clinical trials with exogenous administration of these neurotrophins are disappointing possibly due to a lack of blood–brain barrier permeability, poor half‐life time, and rapid degradation.^[^ ^5^, ^6^ ^]^ We thereby attempt to illuminate if nanomaterials possess abilities to mimic the bio‐activation of neurotrophins, but potentially own long circulation and high affinity catalytic Trk receptors in the brain.

Nanomaterials have a range of versatile physical and chemical properties, but the tunability of the properties for adapting biological systems, especially for neurological disease treatment, is perhaps less well understood. In previous studies, we have utilized the intrinsic activity of single‐walled carbon nanotubes (SWNTs) (but not as drug vector) to treat multiple neuropsychiatric diseases with extremely low dosages (nanogram levels in mice) without neurotoxicity.^[^ ^7^, ^8^ ^]^ In addition, we emphasize that precisely conformational structures of SWNTs determine their functions in neurological diseases.^[^ ^9^, ^10^, ^11^ ^]^ In the current research, we thus first developed a scalable method starting with highly dispersed pristine SWNTs (pSWNTs), which are then separated into three different SWNT structural fractions: individual SWNTs (iSWNTs), bundled SWNTs (bSWNTs), and bundled but defective SWNTs (dSWNTs). Based on these relatively precise nano‐structures, we investigate the therapeutic effect of SWNTs on 4‐day binge ethanol rat model. More importantly, besides the high efficacy, we explain the molecular mechanisms of SWNTs in the treatment of binge alcohol‐induced neurodegeneration.

## 2 METHODS

### 2.1 Drugs

K252a was from Calbiochem (Billerica, MA). BDNF was from R&D Systems (Minneapolis, MN). Recombinant human BDNF was from Novoprotein (Summit, NJ). 7,8‐dihudroxyflavone (7,8‐DHF) was from Tokyo Chemical Industry (Cat. #D1916). All other reagents were from Sigma‐Aldrich (St. Louis, MO) unless otherwise noted.

### 2.2 Animals and binge ethanol exposure treatment

Male Sprague–Dawley rats (56 days, weight 270–300 g) were provided by Changchun Changsheng Life Sciences Limited. The rats were reared under standard conditions (20–24 °C, 40–60% relative humidity, 12 h lighting). All experiments were conducted according to the National Institutes of Health (NIH) Guide for the Care and Use of Laboratory Animals (NIH Publications No. 80‐23, revised 1996). All applicable institutional or national guidelines for the care and use of animals were followed.

Animals were perfused with a full nutritional diet (50% v/v Vanillasure (ABBOTT, Zwolle, Netherlands)) containing ethanol (25%, w/v) through an 18‐gauge gavage needle to subject rats to intermittent ethanol exposure. For neurodegenerative model induced by binge EtOH exposure, rats were given an initial dose of 5 g/kg ethanol (0.2 mL/10 g body weight), and an additional dose of 3 g/kg (0.12 mL/10 g body weight) was given every 8 h thereafter for 4 consecutive days. After 4 days of ethanol exposure, there was a 3‐day ethanol withdrawal period. Control rats received 50% v/v Vanilla Ensure, a diet equal in calories to glucose, which was equal to the average value of all EtOH animals. Based on previous studies, the dosage, duration, and route of administration of ethanol were determined.^[^ ^12^, ^13^ ^]^

### 2.3 Sorting of SWNTs by density gradient ultracentrifugation

Density gradients were formed from aqueous solutions of iodixanol (Opti Prep 60% w/v iodixanol, 1.32 g/cm^3^ (Sigma‐Aldrich, St. Louis, MO)), and the gradient was created step by step in a centrifuge tube. In brief, a layer of 0.2 mL of highly dispersed SWNTs was put on top of a three‐layer water‐iodixanol density gradient (10% + 30% + 60% layers). Centrifugation was carried out in a P40ST swing‐bucket rotor in a Himac CP80WX ultracentrifuge (Hitachi High Technologies, Japan) at 20 °C and centripetal accelerations of about 200 000 g for 12 h. Bands containing separated SWNTs were manually retrieved from the tube for subsequent characterization. Finally, 0.5 mg/mL SWNTs were dispersed into 2 mg/mL sodium deoxycholate, and further diluted with artificial cerebrospinal fluid or culture medium for subsequent experiments.

### 2.4 SWNT administration

Rats were anesthetized with chloral hydrate (350 mg/kg, i.p.), and placed in a stereotaxic frame (51500D, Stoelting, USA). SWNTs or sodium deoxycholate as solvent vehicle were dissolved in 4 μL artificial cerebrospinal fluid. The right lateral ventricle (AP: −0.8 mm, ML: −1.4 mm, DV: −3.6 mm) was injected (0.2 μL/min), and the needle was kept in place for 10 min. To serve as controls (vehicle group), animals in the EtOH group were injected with the solvent sodium deoxycholate, using the same procedure. In sham‐operated rats, scalps only were dissected under anesthetic.

### 2.5 Novel location recognition and novel object recognition

**FIGURE 1:**
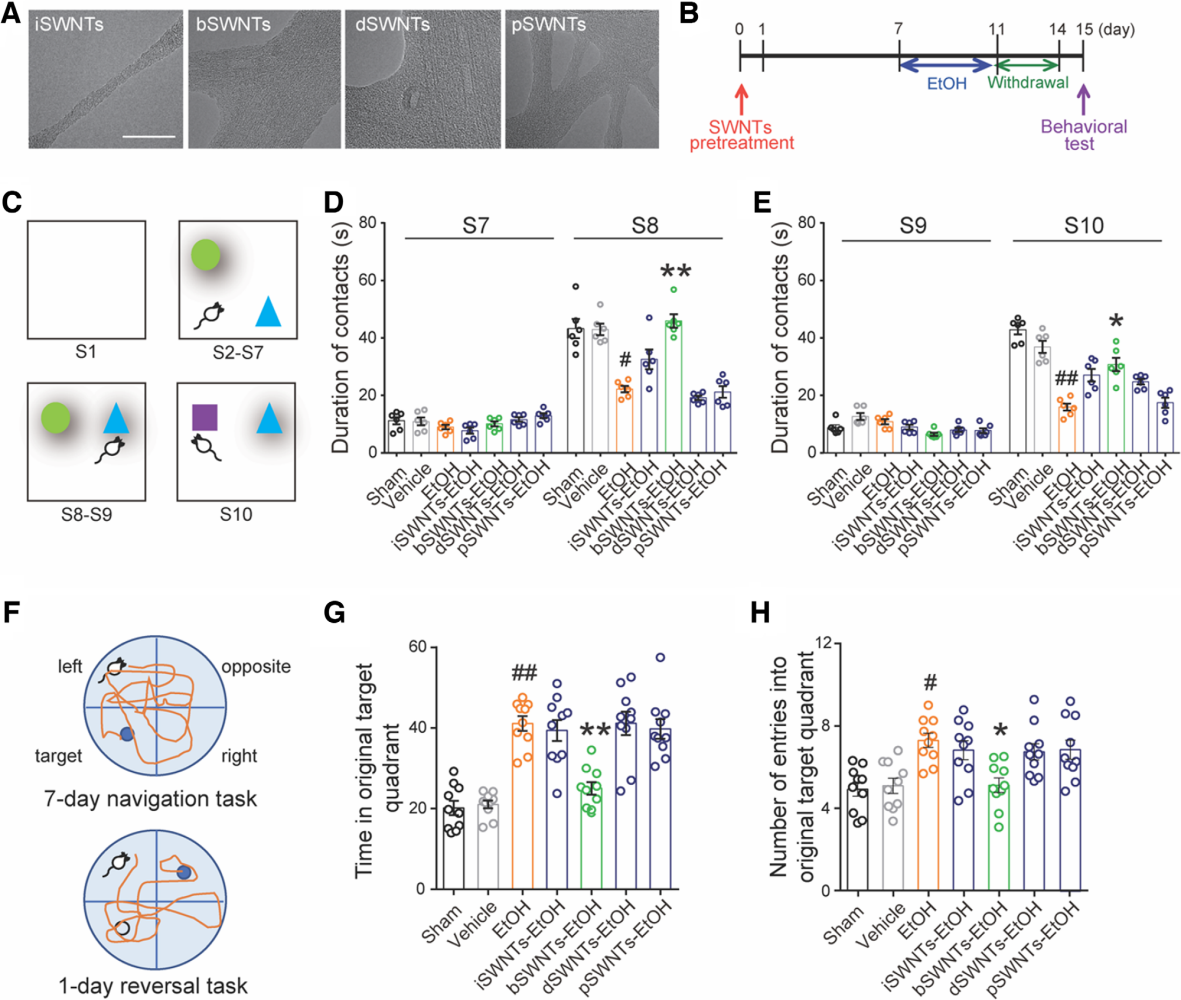
bSWNTs promote the recovery of learning and memory function in rats with EtOH‐induced neurodegeneration. (A) Typical HR‐TEM micrographs of the four SWNTs. Scale bar; 10 nm. (B) Experimental procedure for administration of 4 different types of SWNT (i.c.v. 40 ng/rat). One week before binge ethanol exposure, rats were treated with either vehicle or different SWNTs. Some rats were sacrificed after 4‐day ethanol exposure for subsequent protein analysis, while others underwent behavioral tests after 3 days of withdrawal. (C) Behavioral training schedule for the object recognition tests. (D,E) Representative results from the novel location recognition (D) and novel object recognition (E) tests, indicating the effects of SWNTs on learning and memory. ^#^ *p* < 0.05, ^##^ *p* < 0.01 versus the vehicle group; *p < 0.05, **p < 0.01 versus EtOH group, one‐way ANOVA; *n* = 8. (F) A schematic representation of the Morris water maze protocol. Mice were trained for 7 days in a navigation task to locate a hidden platform in the original target quadrant. The hidden platform was then moved to the opposite quadrant during the reversal task. (G,H) The time spent in the original target quadrant (G) and the number of entries into the original target quadrant (H) in the Morris water maze reversal task. Administration of bSWNTs facilitated the recovery of spatial memory deficits induced by binge ethanol. Data are presented as means ± SEM. ^#^ *p* < 0.05, ^##^ *p* < 0.01 versus the vehicle group; *p < 0.05, **p < 0.01 versus EtOH group, one‐way ANOVA; *n* = 8

Tests were experimented based on previously verified methods.^[^ ^14^ ^]^ Briefly, novel object recognition (NOR) training and testing were conducted in a box (100 cm wide × 45 cm high) with black on all sides and an open top in the uniform environment. The stimulus objects were made of metal, glass, or plastic with distinctive colors and shapes. The test consisted of ten consecutive 4 min sessions, with a 4‐min break after each test (Figure 1C). In session S1, animals were habituated to the experimental arena by allowing them to freely explore the empty open field to prevent anxiety and facilitate assessment of locomotor activity. In the training sessions S2–S7, rats were placed in the field containing a diagonal configuration of two objects 1 and 2, and became habituated to this new environment. Object 2 was relocated to the opposite corner in S8. Rats were placed in the box to explore the objects to test for spatial memory. In S10, object 1 was replaced by a novel object (object 3) in the same location as object 1 to test for recall of object recognition. All objects were at the same distance from the walls of the venue. The recording was carried out by a camera hanging above the field, and EthoVision@3 software (Noldus Information Technology, Wageningen, The Netherlands) was used to connect with the computer tracking system, and calculated the duration of contact. A contact was defined as any time the rat touched an object with its nose or forepaws to actively explore.

### 2.6 Morris water maze test

The Morris water maze test was carried out according to a previously reported method.^[^ ^15^ ^]^ The device consisted of a white circular galvanized steel pool (diameter 160 cm) filled with water (20–21 °C, 2 cm above the surface of the platform) and a 10.2 cm circular platform. Each wall was hanging with different clues outside the maze. The animal's movement in the pool was tracked by video camera and analyzed using EthoVision (Noldus, Wageningen, The Netherlands). The water was stirred between the experiments in each group to destroy the odor traces.

In the navigation task, 7 days of training were carried out to make the animals find and keep on the platform from the water, and each animal was tested 3 times a day. The animal was introduced into the pool, facing the pool wall at each of the three quadrant edges. Once the animal reached the platform, it was allowed to remain on the platform for ∼10 s. The time taken to reach the platform was defined as the escape latency. If the animal failed to reach the platform within the trial ceiling (90 s), it was guided to the platform to remain for 10 s, and was removed to the home cage.

After the navigation task, animals were tested in a reversal‐learning task. The submerged platform was placed in the quadrant opposite that in which it had been placed during the reference memory task (southwest quadrant). Each animal was given three trials lasting 90 s. The animal was introduced into the pool, facing the pool wall at each of the three quadrant edges. Once the animal reached the platform, it was allowed to remain on the platform for approximately 10 s. The escape latency, the time spent in the original target quadrant and the number of entries into the original target quadrant were recorded. If the animal failed to reach the platform within 90 s, it was guided to the platform, where it remained for 10 s. The animal was then removed to its home cage.

### 2.7 Primary neuronal cultures and purification analysis

Dissociated cortical primary neurons were prepared from E16 rats. After dissection, neurons were maintained at 37 °C with a 5% CO_2_ atmosphere in neurobasal medium containing B27 (Invitrogen, Rockville, MD) as previously described. SWNTs were added 24 h after 6‐day culture, and the cell viability was assayed by CCK‐8 (CK04, Dojindo Laboratories, Japan). For the in vitro cytotoxicity test, neurons were plated in 96‐well plates with ∼85% confluency. Primary neurons were treated with vehicles, EtOH or bSWNTs for 24 h, then CCK‐8 assay was used to examine the cell viability.

### 2.8 Immunocytochemistry and quantification of axon growth

Neurons were plated on 35 mm glass‐bottom chamber dishes. After treatment, neurons were fixed with 4% paraformaldehyde for 15 min at room temperature (RT), then blocked with 10% BSA, and incubated with Tuj‐1 (1:1000, Novus Biologicals, St. Charles, MO) at 4 °C overnight. Cy2‐labeled secondary antibody (1:500, Novus Biologicals, St. Charles, MO) was added for 1.5 h at RT, and then slides were stained with DAPI (Invitrogen, Rockville, MD) to identify nuclei.

Treated cultures were immunolabeled for neurofilaments and images were obtained using confocal microscopy (Leica, DMI6000B). Image acquisition and subsequent analysis of 30 randomly separated cells from each group was performed by investigators blinded to the experimental conditions. Five separate culture repeats were analyzed.

Intraperitoneal injection of chloral hydrate (350 mg/kg) to completely anesthetized the animal, then the brains were fixed by transcardial‐perfusion with saline solution containing heparin (10 U/mL), followed by perfusion and immersion in 4% paraformaldehyde dissolved in 0.1 M phosphate buffer solution (pH 7.4). After post‐fixation for 3 h, the brains were transferred to 30% sucrose. 20 μm‐thick sections were cut from the block on a freezing microtome (AS‐620, Shandon, Asomoor, UK). Every sixth section was collected on coated slides and four sets of slides were obtained equally for immunohistochemistry analysis. Briefly, sections were washed in tris‐buffered saline (TBS) then incubated in 0.3% H_2_O_2_ for 30 min. Following TBS washes, sections were incubated in 5% normal goat serum (containing 0.1% Triton‐X/TBST) for 60 min.

For DCX and BrdU labeling, sections were pre‐treated with 0.01 M citrate buffer (pH 6.0) in a 85 °C steamer for 25 min, and then allowed to cool to RT for 20 min. Sections were treated with 2 N HCl at 37 °C for 20 min, then incubated in 5% normal goat serum before treatment with primary (anti‐BrdU, 1:400. Abcam ab20715; anti‐DCX, 1:300, Abcam, ab6326) and secondary antibodies (Donkey anti‐rat Alexa 405 and Donkey anti‐rabbit Alexa 555, 1:400).

### 2.9 Fluoro‐Jade B staining

Fluoro‐Jade B (FJB) (Millipore, Billerica, MA) staining was performed on adjacent 20 μm sections according to the manufacturer's protocol. FJB‐positive (FJB+) cells were photographed with a fluorescent microscope (DMI3000a, Leica, Stockach, Germany) fitted with a FITC filter set and a digital camera DP71. Image analysis was performed using an Image‐Pro 3D Plus Workstation for Fluorescence Imaging Processing (Media Cybernetics, Rockville, MD).

### 2.10 Transmission electron microscopy

Animals were sedated with an overdose of pentobarbital and perfused transcardially with 0.1 M phosphate buffer and fixative (2% paraformaldehyde/2.5% glutaraldehyde in 0.15 M sodium phosphate buffer). Representative areas were dissected from the brain and postfixed in 1% osmium tetroxide/1.25% potassium ferrocyanide in 0.15 M sodium phosphate buffer for 1 h. Samples were dehydrated in a graded ethanol series, followed by propylene oxide, infiltrated and embedded in Polybed 812 resin (Polysciences, Inc., Warrington, PA). Seventy‐nanometer ultrathin sections were taken, mounted on 200 mesh copper grids, stained with uranyl acetate and lead citrate. Sections were observed and photographed using an HT7700 transmission electron microscope (Hitachi High Technologies, Japan) at an accelerating voltage of 80 kV.

### 2.11 Western blots

Animals were sacrificed after 4 days of binge ethanol exposure. Brains were harvested on ice and homogenized in RIPA lysis buffer (Beyotime, Shanghai, China). Each sample was a mixture of hippocampal and prefrontal cortex tissue from at least four rat brains. Brain tissue protein extracts were separated by SDS‐PAGE, transferred to nitrocellulose membranes (Millipore, Billerica, MA) and probed with the following primary antibodies: α‐tubulin (1:1000), β‐Actin (1:1.000), PI3K (1:200) (Santa Cruz Biotechnology, CA); p‐ERK and total ERK, p‐Akt and total Akt, p‐TrkA and TrkA, p‐TrkB and TrkB (1:1000, Cell Signaling Technology, Danvers, MA); doublecortin antibody 3E1 (DCX), tubulin‐β‐III (Tuj‐1,1:1000, Novus Biologicals, St. Charles, MO), microtubule‐associated protein 2 (MAP2, 1:1000, Synaptic Systems, Gottingen), and BDNF (1:500, Abcam). Blots were then probed with appropriate HRP‐conjugated secondary antibodies (Santa Cruz Biotechnology, CA), and immunoreactive proteins were detected using the ECL detection system (Thermo, Rockford, IL). Western blot analysis was performed on samples from three separate experiments and each sample was a mixture of tissues from four rat brains per group. Band optical densities were quantified with Image J software (NIH, MD).

### 2.12 Fluorescence resonance energy transfer measurements

HEK293 cells were transfected with TrkB‐CFP (donor) and TrkB‐YFP (acceptor) at 50% confluence, then treated with 7,8‐DHF or bSWNTs for 30 min. The fluorescence resonance energy transfer (FRET) experiment was performed with a confocal microscope (Leica, DMI6000B). FRET was determined by pixel‐by‐pixel calculation using apparent FRET efficiency (Eapp) calculation methods.^[^ ^16^ ^]^ *A*, *B*, and *C* correspond to the intensities of the three signals (donor, FRET, acceptor) and *α*, *β*, *γ*, and *δ* are the calibration factors generated by acceptor only and donor only references. Eapp value was calculated according to the following equation: Eapp = (*B* − *A* × *β* − *C* × *γ*)/*C*.

### 2.13 Surface plasmon resonance

Surface plasmon resonance (SPR) was performed on a PlexArray HT System. Briefly, TrkA, TrkB, and TrkC (R&D Biotechnology, Minneapolis, MN) were immobilized (at 50 μM) on an Au chip, then blocked with aminoethanol. Running buffer was 10 mM HEPES, 150 mM (NH_4_)_2_SO_4_, 1.5 mM CaCl_2_, 1 mM EGTA, 0.005% v/v Tween‐20, pH 7.4. bSWNTs were applied at 100 μg/mL, and the sensor chip was regenerated in a 10 mM glycine‐HCl buffer after each analytic cycle. The SPR signal was expressed in relative response units as the response obtained in a control flow channel was subtracted.

### 2.14 Plasmids and cell transfection

Full‐length TrkB cDNA was inserted into pEGFP‐N1, pECFP‐N1, pEYFP‐N1, and Myc‐pCMV vectors (Clontech) to generate TrkB‐GFP, TrkB‐CFP, TrkB‐YFP, and Myc‐TrkB by In‐fusion procedure (Clone smarter, PC891). Human TrkB was amplified by PCR and cloned into pEGFP‐N1 between XhoI and BamHI sites, pECFP‐N1 and pEYFP‐N1 between HindIII and BamHI sites, and Myc‐pCMV between EcoRI and KpnI sites. HEK293 cells were transfected with plasmids using Lipofectamine 2000 (Life Technologies, 12566014) for 24 h according to the manufacturer's instructions.

### 2.15 Fluorescence spectrometry

HEK293T cells were transfected with GFP‐TrkB plasmids for 24 h. After that, the cell culture solution was discarded, and the PBS was washed 3 times for 5 min every time. The cells were lysed by protein lysate, centrifuged at 13 400 rpm for 10 min, and the supernatant was collected. bSWNT was added to the collected supernatant, and its fluorescence intensity (EX: 488 nm; EM: 509 nm) was measured by fluorescence spectrophotometer (Hitachi, f‐7000, Japan).

### 2.16 Fluorescence quenching experiment

We observed the binding of bSWNT to GFP‐TrkB in living cells. HEK293T cells were transfected with GFP‐TrkB plasmids for 24 h. The fluorescence changes of GFP‐TrkB in living cells were observed by fluorescence confocal microscopy (Leica, TCS SP8, Germany) under 40 times oil mirror (EX: 488 nm; EM: 509 nm). After continuous observation for 30 s, the image was intercepted every 10 s. After bSWNT treatment, the fluorescence of GFP‐TrkB was dynamically quenched.

### 2.17 TrkB knockdown in rats

Three shRNA sequences for rat TrkB were inserted into the AAV vector. The recombinant AAV2/9 expression cassette was ITR‐CAG‐EGFP‐micro30 shRNA TrkB‐WPRE‐hGH polyA‐ITR (ITR, inverted terminal repeat; CAG, chicken β‐Actin promoter modified with CMV early enhancer; WPRE, cis‐acting woodchuck post‐transcription regulatory element; hGH polyA, human growth hormone transcription termination sequence). The shRNA sequences were:

TrkB shRNA1: 5′‐TTGTGGATTCCGGCTTAAAGTAGTGAAGCCACAGATGTACTTTAAGCCGGAATCCACAA‐3′
TrkB shRNA2: 5′‐GGATGACAGTGGGAAACAAATAGTGAAGCCACAGATGTATTTGTTTCCCACTGTCATCC‐3′
TrkB shRNA3: 5′‐GGAGTTGACTATGAGACAAATAGTGAAGCCACAGATGTATTTGTCTCATAGTCAACTCC‐3′

The resultant AAV‐CAG‐EGFP‐shRNA TrkB (AAV2/9) was packaged at Obio Technology Company (Shanghai, China). The shRNA knockdown ability was tested 11 days after adding the viruses to primary cultured neurons (multiplicity of infection 104). The packaged AAV were concentrated in PBS at the following titers: AAV‐shScramble 6.6 × 1012 v.g./mL; AAV‐shTrkB1 3.11 × 1012 v.g./mL; AAV‐shTrkB2 2.97 × 1012 v.g./mL, AAV‐shTrkB 3 × 1012 v.g./mL. All viruses were diluted to 2 × 1012 v.g./mL with PBS before virus injection.

Stereotactic delivery of the AAV vectors into the hippocampus was performed after the rats were anesthetized with chloral hydrate. Briefly, 2 μL of AAV particles were injected into the hippocampus (A/P – 3.72 mm, M/L ± 2.2 mm, D/V −3.7 mm from bregma). The injection was performed by inserting a 33G Hamilton auto‐injector at a speed of 0.25 μL/min. To prevent reflux, the needle was kept in place for 10 min, withdrawn a short distance, and then placed in the new position for 5 min before removal. Behavioral experiments were performed after the rats recovered for 4 weeks.

### 2.18 GFP‐trap assay

After transfection with TrkB‐GFP and Myc‐TrkB, cells were treated with 7,8‐DHF or SWNTs for 30 min. Cells were washed 3 times with PBS and lysed with lysis buffer (50 mM Tris‐HCl pH 7.5, 150 mM NaCl, 1 mM EDTA, 1% Triton X‐100, and protease inhibitor cocktail) for 30 min. Lysates were centrifuged for 15 min at 4 °C at 13400 rpm, and the supernatant (input) was collected.

Lysates were immunoprecipitated by 25 μL pre‐cleaned GFP‐Trap agarose beads (V‐nanoab, GNA‐20‐400) at 4 °C for 1 h. After centrifugation and 4 washes in 1 × TBS + 0.1% NP‐40, the beads were collected. 2 × SDS‐PAGE sample buffer was added and the samples were boiled for 10 min at 95 °C for immunoblotting. The antibodies were: anti‐Myc (1:1000, Sigma, M5546), anti‐GFP (1:2000, EASYBIO, 80931106).

### 2.19 In situ proximity ligation assay

HEK293 cells were transfected with Myc‐TrkB at 50% confluence, then treated with 7,8‐DHF and bSWNTs for 30 min. The samples were fixed with 4% paraformaldehyde for 15 min at RT. After blocking with Duo‐link blocking solution (Sigma, DUO82007) at 37 °C, samples were incubated for 1 h with primary antibodies (1:400, Sigma, M5546; 1:200, CST, #2278) and incubated with proximity ligation assay (PLA) Probe (DUO82004) for 1 h at 37 °C. Follow the manufacturer's instructions for the experiment and slides were mounted with a coverslip using a minimal volume of Duolink In Situ Mounting Medium with DAPI (DUO82040). Fluorescent images were acquired by a Leica confocal microscope (DMI6000).

### 2.20 Gene microarray analysis

Total RNA was extracted and the gene library was constructed. The Agilent 2100 Bioanalyzer and ABI StepOnePlus Real‐Time PCR System were used for quality inspection and then sequenced (BGI, China). Gene expression cluster analysis, gene co‐expression network analysis and differential function gene GO function analysis were performed according to the manufacturer's recommendations.

### 2.21 Statistical analysis

All data are expressed as mean ± SEM. The density of immunoblot bands was quantified using Image J software (NIH Image). Statistical significance was determined using one‐way or two‐way ANOVA. Dunnett's multiple comparison tests were used to analyze the between‐group differences.

## 3 RESULTS

### 3.1 Preparation of four SWNT structures

The efficiency of density gradient ultracentrifugation (DGU) of SWNTs in aqueous solution was affected by their density, surface modification, and thickness of the electrostatically bonded hydration layer. After being separated by DGU, atomic force microscopy images were used to compare diameter, length, aggregation and structural integrity of the starting material (pSWNTs) and the three DGU fractions (iSWNTs, bSWNTs, and dSWNTs) (Figure S1A). The iSWNT fraction contained individually dispersed SWNTs with an average diameter of 1 nm, while the bSWNTs and dSWNTs contained aggregated bundles with average diameters of 15–20 nm. As further revealed by the HR‐TEM measurements, the dSWNT fraction possessed fragments with poor structural integrity (Figure 1A). The length distribution (Figure S1B), the visible‐near infrared absorption spectra (Figure S1C), and Raman spectra (Figure S1D) of the four SWNTs are consistent with previous studies, which suggested our SWNTs are generally easy to separate with uniform and stable characterizations.^[^ ^9^, ^17^ ^]^

### 3.2 bSWNTs restore learning and memory of binge‐drinking rats

We then extended our study of different types of SWNT by testing them in the 4‐day binge ethanol rat model, a representative model of NDD.^[^ ^14^, ^18^ ^]^ It is well documented that memory loss and cognitive dysfunction occur in rats after a 4‐day EtOH binge, which directly damages important areas of the brain, including the hippocampus and prefrontal cortex.^[^ ^19^ ^]^ We singly injected different types of SWNT (40 ng/rat) or equivalent vehicle to the lateral ventricle of Sprague–Dawley rats (intracerebroventricular injection, i.c.v., Figure S2) 1 week before 4‐day binge EtOH exposure (Figure 1B). The results showed that none of the SWNTs affected the locomotor activity (Figure S3). After that, we observed the effects of the four types of SWNT on object recognition, a classical test for studying recognition memory in animals.^[^ ^20^ ^]^ During each test session, animals explored an enclosed space that contained two objects in two different positions (novel location recognition, NLR) or with different shapes, textures, and colors (NOR) (Figure 1C). In the NLR test, we observed no significant changes between sham‐operated and vehicle‐operated rats, indicating that the equivalent vehicle of SWNTs could not influence the neurobehaviors of rats. Moreover, we found that the time spent exploring the re‐located object in the EtOH‐treated group was significantly less than that in sham‐operated and vehicle‐treated groups, indicating that EtOH treatment could significantly induce memory impairments in rats (Figure 1D). However, compared with binge ethanol exposure group, the bSWNT‐treated group took much more time in exploring the re‐located object (Figure 1D). Similarly, in the NOR test (sessions 9–10), bSWNT‐treated animals spent significantly longer exploring the novel object than the EtOH‐treated rats (Figure 1E). The other SWNTs had minimal effects on the performance in the NLR or NOR tests. We also used the Morris water maze to assess relearning ability (Figure 1F).^[^ ^15^ ^]^ During the 7‐day navigation task, animals were allowed to locate a hidden platform in a tank of water and the time spent to find the platform was defined as the escape latency. No difference was found in escape latency among the different groups (Figure S4). The rats were subsequently given a reversal task in which the hidden platform was moved to the opposite quadrant in the Morris water maze tank (Figure 1F). However, during the reversal task, the time in the original quadrant (Figure 1G) and the number of entries into the original quadrant (Figure 1H) in the EtOH‐treated group were significantly greater than in the sham‐operated and vehicle‐treated groups. In contrast, the bSWNT‐treated animals spent significantly less time in the original quadrant and made fewer entries than the binge ethanol exposure group. Therefore, the behavioral data showed that compared to other SWNTs, bSWNTs is the most efficient components to improve the recovery of memory function in rats with binge EtOH exposure.

### 3.3 bSWNTs possess functions of neuroprotection and neuroregeneration

**FIGURE 2:**
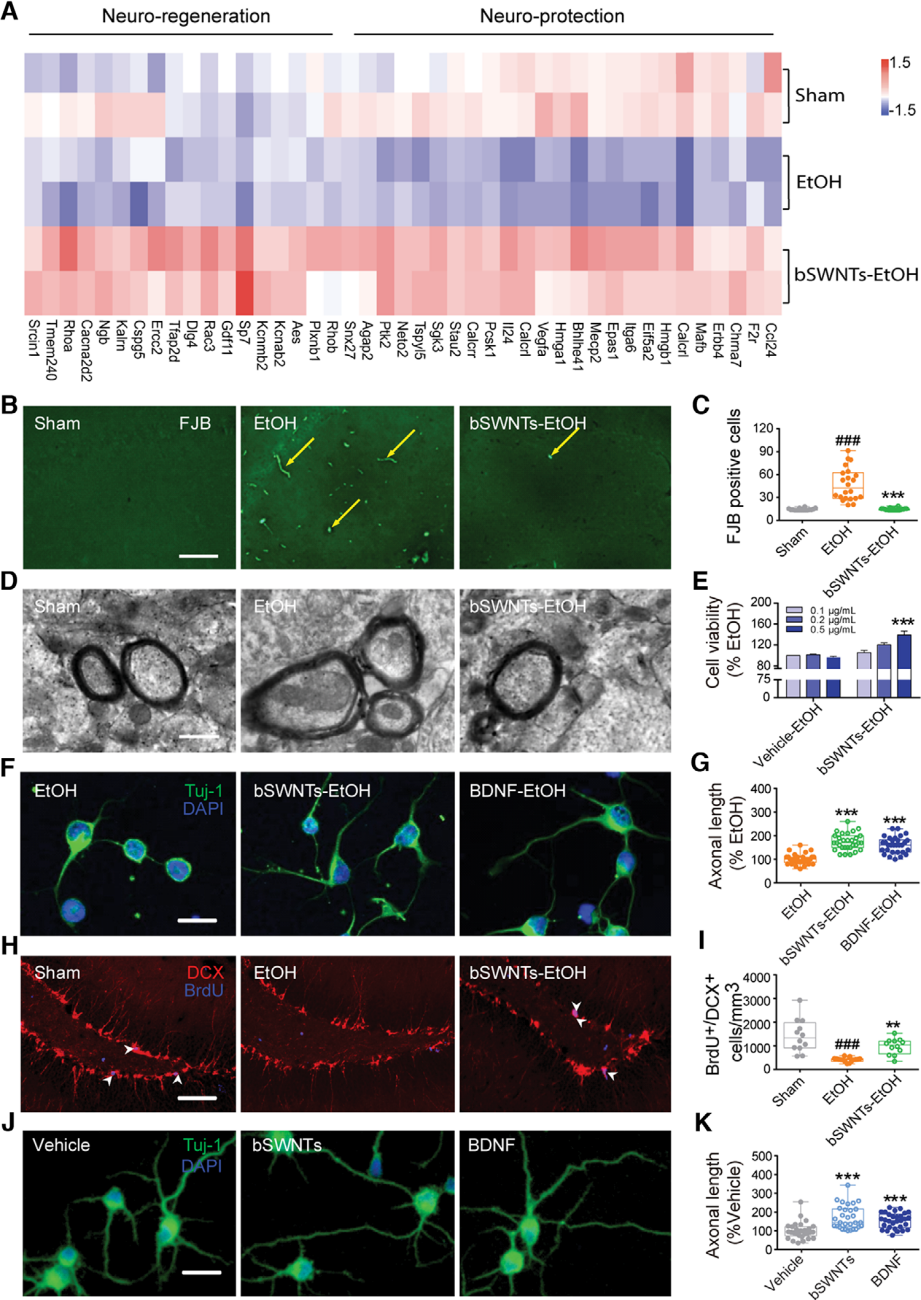
bSWNTs protect neurons from EtOH‐induced death, and stimulate neuronal growth and regeneration. (A) Heatmaps of gene‐expression data from microarray analysis. Global gene expression changes were measured by high‐throughput sequencing analysis, and genes associated with neuro‐protection and neuro‐regeneration were calculated in sham, EtOH and bSWNT‐administrated groups. The data shown represent two independent experiments, and each sample contained hippocampus from two rats. (B) Photomicrographs of the hippocampal region showing degenerating neurons, which are stained by FJB as bright green puncta (yellow arrows). Fewer FJB‐positive cells are seen in the bSWNT administration group than in the EtOH group. Scale bar, 100 μm. (C) Quantitative analysis of FJB^+^ cells in the hippocampus. Data are presented as the mean number of FJB‐positive cells/mm^2^ ± SEM. ^###^ *p* < 0.001 versus the sham group; ***p < 0.001 versus the EtOH group; *n* = 16 sham, *n* = 22 EtOH, *n* = 22 bSWNTs‐EtOH from 4 rats. (D) TEM showing the ultra‐structure of EtOH‐induced changes in neuronal myelin, which were reversed by bSWNTs. Scale bar, 20 nm. (E) Cell viability assay by CCK‐8 showing that bSWNTs protected neurons from EtOH‐induced primary neuron death. Data are presented as means ± SEM. ***p < 0.001 versus the EtOH group. (F) Confocal images of EtOH‐damaged neurons treated with bSWNTs, and BDNF as the positive control. Scale bar, 20 μm. (G) Bar graphs showing quantifications of axonal length in panel (F) by Image J. Data are presented as means ± SEM; ***p < 0.001 versus the EtOH group; *n* = 30 from 5 independent repeats. (H,I) Confocal images and quantification of DCX/BrdU immunostaining in hippocampus. White arrowheads indicate colocalization of DCX/BrdU staining. Scale bar, 50 μm. Administration of bSWNTs caused a significant increase in the number of DCX^+^/BrdU^+^ cells. Data are presented as means ± SEM. ^###^ *p* < 0.001 versus the vehicle group; **p < 0.01 versus the EtOH group. *n* = 15 EtOH group, others *n* = 12 from 3 rats per group. (J) The morphology of cortical primary neurons stained by Tuj‐1 and DAPI. (K) Quantitative analysis of the axonal length, showing the significant increase caused by treatment with bSWNTs. BDNF treatment was a positive control. Data are presented as means ± SEM. ***p < 0.001 versus the vehicle group. *n* = 30 cells from 5 independent cultures

To reveal the possible functions of bSWNTs for binge alcohol treatment, analysis of a broad‐spectrum gene screening was performed. We noticed that multiple up‐regulated or down‐regulated genes were highly involved in neuroprotective effects and neurogenesis promotion within the bSWNTs group (Figure 2A). Then we first demonstrate whether the impact of bSWNTs is mediated by preventing brain damage in vivo. In EtOH‐treated rats, FJB staining revealed substantial neuronal cell death in the hippocampus (Figure 2B,C) and prefrontal cortex (Figure S5), which was rescued by pretreatment with bSWNTs. The expression of MAP2, a neuronal marker reduced by binge EtOH, was also augmented by treatment with bSWNTs (Figure S6). Disordered and less compact myelin sheaths are also a symptom of EtOH‐induced NDDs.^[^ ^14^, ^21^ ^]^ After pretreatment with bSWNTs, the myelin sheaths exhibited a regular, healthy appearance (Figure 2D). Also, bSWNTs prevented the death of primary cultured cortical neurons (Figure 2E) and markedly decreased EtOH‐induced axonal damage in vitro, which is similar to BDNF, the endogenous ligand of TrkB (Figure 2F,G).

Several studies have illustrated that carbon nanotubes might stimulate neural growth and cellular neurogenesis as supporting materials.^[^ ^22^, ^23^ ^]^ However, the possible mechanisms have not been carefully explored. We thus monitored whether neurogenesis‐related activities underlie the above effects of SWNTs. We observed that bSWNTs significantly upregulated DCX expression (Figure S7), an indicator of neuroblasts and developing neurons, suggesting that bSWNTs may exert their protective effects against EtOH‐induced damage by generating new‐born neurons. The number of proliferating neurons, showing positive for both DCX and the proliferation marker BrdU in the hippocampus, was significantly increased in the bSWNT‐treated group compared to the EtOH group (Figure 2H,I). Furthermore, bSWNTs directly stimulate the axonal growth of primary cultured cortical neurons (Figure 2J,K). Taken together, these results confirmed the ability of bSWNTs to promote neurogenesis in rats with EtOH‐induced neurodegeneration.

### 3.4 bSWNTs directly interact with neurotrophic receptor as a potential target

**FIGURE 3:**
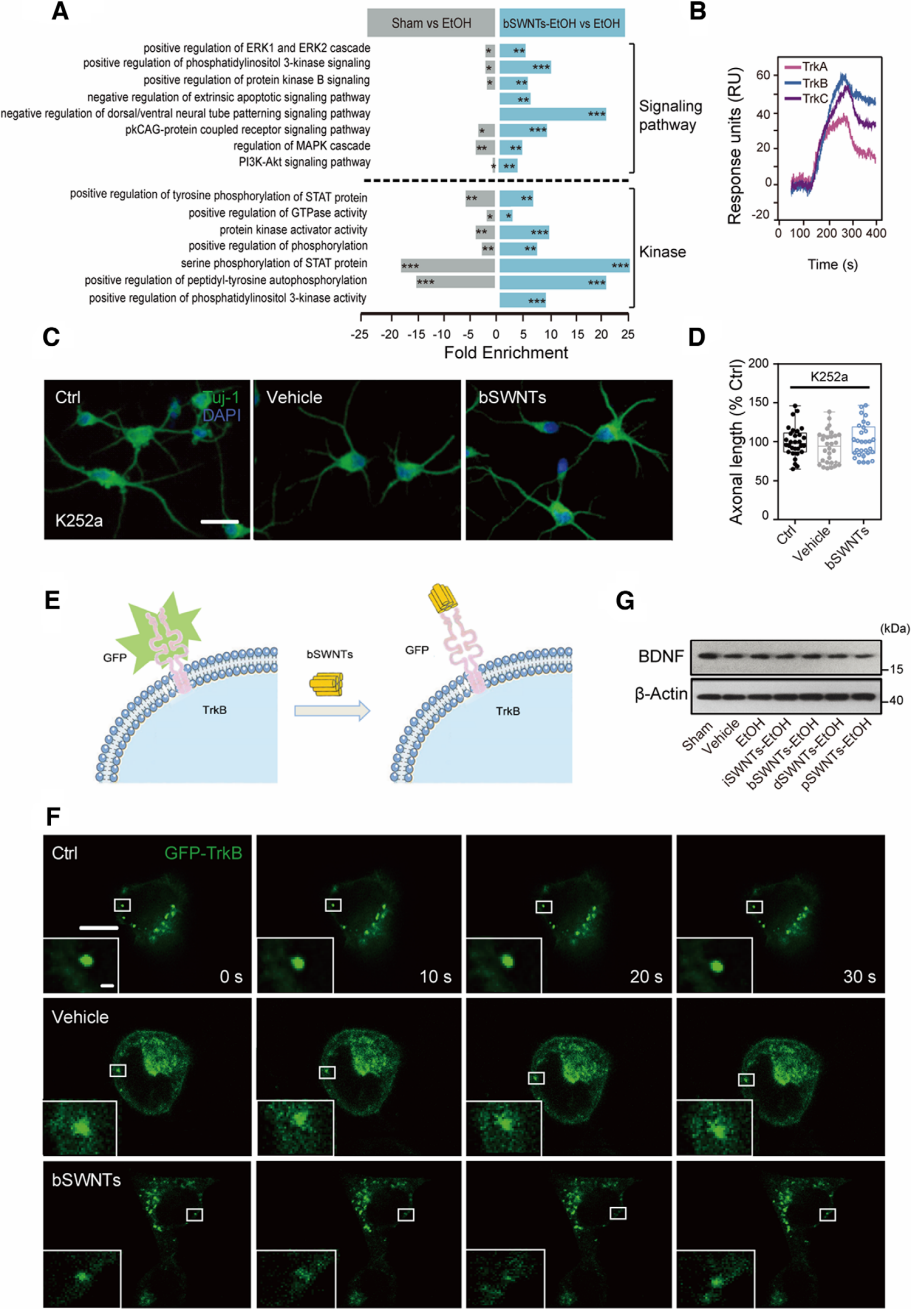
TrkB is a potential target of bSWNTs for neuronal protection and regeneration. (A) Selective GO function enrichment analysis significantly upregulated in bSWNTs‐treated group. Functional terms showing bSWNTs mainly affected MAPK and Akt/PI3K signaling pathways, and kinase activity/phosphorylation were screened out in EtOH versus Sham, and bSWNTs‐EtOH versus EtOH groups. The fold enrichment is expressed from −25 to 25. *False discovery rate (FDR)‐adjusted *p* ≤ 0.05, **FDR‐adjusted *p* ≤ 0.01, ***FDR‐adjusted *p* ≤ 0.001. (B) SPR assay demonstrating the interaction between bSWNTs and full‐length TrkA, TrkB, or TrkC. (C) Confocal images of the enhancement of axonal growth by bSWNTs, which is inhibited by the TrkB antagonist K252a. Scale bars, 20 μm. (D) Quantitative data from (C). Bar graphs show quantifications of axonal length by Image J. Data are presented as means ± SEM. *n* = 30 cells from 5 independent cultures. (E) Schematic diagram of GFP‐TrkB binding to bSWNT. (F) Continuous confocal imaging recording quenched fluorescence of GFP‐TrkB when directly binding with bSWNTs, suggesting bSWNTs dynamically recruit to GFP‐TrkB. Scale bar, 20 μm. (G) The expression of BDNF in hippocampus was detected by immunoblotting. No significant changes were observed after different types of SWNT treatment

The excellent efficacy of bSWNTs in treating NDD prompted us to identify their potential targets. Compared with the EtOH group, the kinase‐related gene expression in bSWNTs‐treated group was upregulated; Moreover, the enriched genes of the MAPK and PI3K/Akt pathways were detected in bSWNTs‐treated group (Figure 3A). Accompanied with the neuroprotective and neurogenesis functions of bSWNTs we explored above, we predicted that Trk receptors (a family of three tyrosine kinase proteins that regulate neuronal growth and genesis via the above signaling pathway) might be probably the targets for bSWNTs. We then detected the possible interactions between bSWNTs and Trk receptors in vitro and in vivo. SPR analysis showed that bSWNTs can bind with the three most common Trk receptors, TrkA, TrkB, and TrkC, but bSWNTs had higher affinity toward TrkB than TrkA or TrkC (Figure 3B). When treated with the specific Trk receptor inhibitor K252a,^[^ ^24^, ^25^, ^26^ ^]^ the neuronal growth induced by bSWNTs was completely blocked, suggesting the efficacy of bSWNTs may be via TrkB receptor (Figure 3C,D). To verify if bSWNTs were directly interacted with TrkB or regulated it bypass, we labeled TrkB with GFP tag, and a clear quenching effect occurred when TrkB‐GFP was adsorbed by bSWNTs (Figure S8). A schematic representation of the direct binding of GFP‐TrkB to bSWNT (Figure 3E), indicating that bSWNT binds to the TrkB on the plasma membrane and quenches green fluorescence. When treated with bSWNTs in live cells, the fluorescence of GFP‐TrkB was dynamically quenched when directly binding with bSWNTs, suggesting bSWNTs recruit to GFP‐TrkB (Figure 3F). Moreover, we observed no significant changes in the protein expression of BDNF when different types of SWNTs were administered, indicating that the therapeutic effect of bSWNTs was not achieved by adjusting BDNF, the endogenous ligands of TrkB (Figure 3G and Figure S9).

### 3.5 bSWNTs lose their therapeutic effects by TrkB depletion in vivo

**FIGURE 4:**
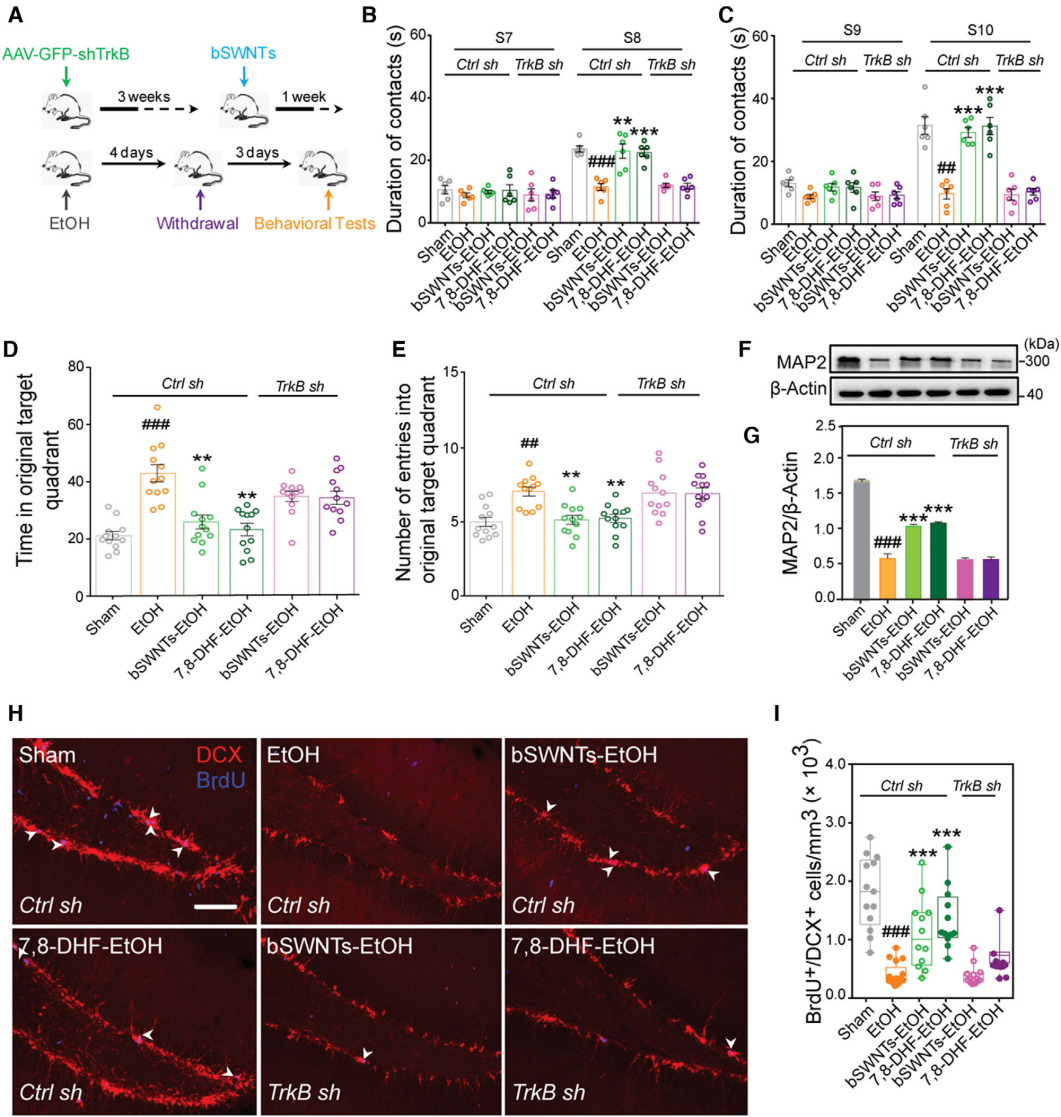
bSWNTs improve neurological function by TrkB. (A) Graphic illustration showing the timeline of TrkB knockdown, administration of bSWNTs, EtOH treatment and withdrawal, and behavioral tests. (B,C) Results from the novel location recognition test (B) and the novel object recognition test (C) to determine the effects of SWNTs and the specific TrkB agonist 7, 8‐DHF on learning and memory improvement in control, and TrkB knockdown rats. Data are presented as means ± SEM. ^##^ *p* < 0.01, ^###^ *p* < 0.001 versus the sham group; **p < 0.01, ***p < 0.001 versus EtOH group, one‐way ANOVA; *n* = 6. (D) Time in the original target quadrant in the Morris Water maze behavioral test, and (E) number of entries into the original target quadrant in the Morris water maze test, indicating that bSWNTs lost their ability to enhance memory recovery after TrkB knockdown. Data are presented as means ± SEM. ^##^ *p* < 0.01, ^###^ *p* < 0.001 versus the sham group; **p < 0.01 versus EtOH group, one‐way ANOVA; *n* = 8. (F) MAP2 expression detected by western blotting after TrkB knockdown. (G) Quantification of MAP2 expression in hippocampus. Data are presented as means ± SEM; ^###^ *p* < 0.001 versus the sham group; ***p < 0.001 versus the EtOH group; *n* = 4 from 3 rats. (H) Double staining of DCX^+^/BrdU^+^ cells in hippocampus. White arrowheads indicate colocalization of DCX/BrdU staining. Scale bar, 50 μm. (I) Quantitative results from (H) indicating that TrkB knockdown also blocks the neural regeneration induced by administration of bSWNTs or 7,8‐DHF. Data are presented as means ± SEM. ^###^ *p* < 0.001 versus the sham group; ***p < 0.001 versus EtOH group; *n* = 13 sham, *n* = 17 EtOH, *n* = 12 bSWNTs‐EtOH, *n* = 13 7,8‐DHF‐EtOH under Ctrl sh; *n* = 12 bSWNTs‐EtOH, *n* = 12 7,8‐DHF‐EtOH under shTrkB knockdown, from 5 rats per group

To further verify if TrkB is the target of bSWNTs and involved in the therapeutic effects, we knocked down TrkB in the hippocampus of rats (Figure S10). Figure 4A shows the timeline of the gene knockdown and subsequent experiments. First, the locomotor activity was not affected by either shTrkB knockdown or administration of bSWNTs (Figure S11). In object recognition tests (Figure 4B,C) and the Morris water maze (Figure 4D,E and Figure S12) tests, although animals treated with bSWNTs and the TrkB agonist 7,8‐DHF clearly showed recovery of learning and cognitive abnormalities, loss of function of TrkB completely blocked these behavioral improvements.^[^ ^6^ ^]^ The increased levels of MAP2 expression (Figure 4F,G) and the higher number of BrdU^+^/DCX^+^ cells in hippocampus (Figure 4H,I) illustrated that the therapeutic effects of bSWNTs on neuroprotection and neurogenesis are dependent on TrkB functions.

### 3.6 bSWNTs stimulate the conformational changes of TrkB protein

**FIGURE 5:**
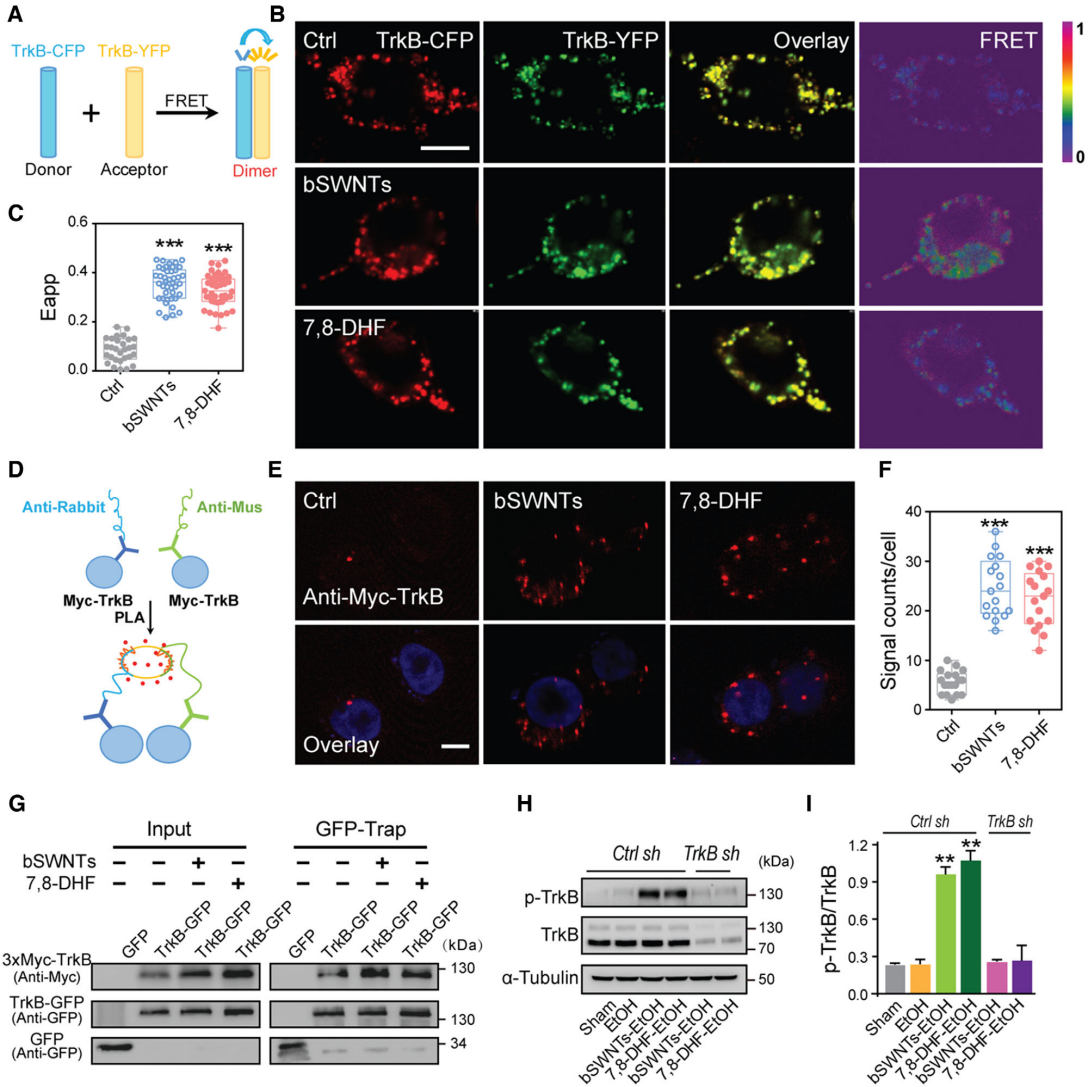
Mechanisms of TrkB activation by bSWNTs. (A) Illustration of the FRET experiment. (B) FRET signal was measured between TrkB‐CFP and TrkB‐YFP in HEK293 cells. Scale bar, 10 μm. (C) Quantification of the apparent FRET efficiency (Eapp) from panel (B). Data are presented as means ± SEM. ***p < 0.001 versus Ctrl group. Three random regions were selected in each counted cell. *n* = 29 Ctrl, *n* = 42 bSWNTs, *n* = 45 7,8‐DHF. (D) Schematic showing the Duo‐link PLA to detect the TrkB‐TrkB interaction. (E) Red spots representing individual TrkB dimerization events were visualized by the PLA. Scale bar, 10 μm. (F) Quantification of red spots in the PLA assay presented in the panel (E). Data are presented as means ± SEM. ***p < 0.001 versus Ctrl group; *n* = 18 Ctrl, *n* = 17 bSWNTs, *n* = 17 7,8‐DHF. (G) In a GFP‐Trap assay, Myc‐TrkB was precipitated by TrkB‐GFP from HEK293 cells treated with bSWNTs or 7,8‐DHF, which indicates that these treatments promote TrkB dimerization. (H) Western blots illustrating that TrkB knockdown suppressed the bSWNT‐ or 7,8‐DHF‐stimulated expression and phosphorylation of TrkB. (I) The ratio of phospho‐TrkB and total TrkB was increased after administration of bSWNTs or 7,8‐DHF, but was dramatically decreased by shTrkB. Data are presented as means ± SEM. **p < 0.01 versus EtOH group; *n* = 4 rats per group

We then illustrated how bSWNTs affect the conformation of TrkB protein and their functions. We found that bSWNTs induced robust FRET signals between TrkB‐CFP and TrkB‐YFP (Figure 5A,B), as measured by FRET Eapp values (Figure 5C). These results indicate bSWNTs stabilize the dimerization of TrkB. We also performed an in situ PLA (Figure 5D) to visualize whether bSWNTs promoted and/or stabilized TrkB dimerization. We observed more Trk‐dimer‐positive dots in the bSWNTs group compared with the control group (Figure 5E,F). In a Co‐IP (GFP‐Trap) assay, bSWNTs increased the precipitation of TrkB, and the effect of bSWNTs on stimulating TrkB dimerization was approximately equivalent to that of the TrkB agonist 7,8‐DHF (Figure 5G). Furthermore, bSWNTs exhibited the most potent in this assay compared with other types of SWNT (Figure S13), which further supported that bSWNTs are the most effective in treating binge drinking.

Next, the level of TrkB phosphorylation in brain tissue was examined. In animals treated with bSWNTs, there was a remarkable increase in the ratios of phospho‐Trk to complete forms of TrkB compared to the EtOH‐treated group. Loss of function of TrkB suppressed the bSWNT‐ or 7,8‐DHF‐induced TrkB phosphorylation, suggesting that bSWNTs acted as a specific inducer of TrkB (Figure 5H,I and Figure S14). Previous studies have clearly shown that BDNF, as a ligand for TrkB, plays a structural role in triggering TrkB dimerization and autophosphorylation, which results in the activation of downstream signaling pathways including the PI3K/Akt and MAPK pathways.^[^ ^27^, ^28^ ^]^ These evidences clearly explain the behavior of bSWNTs to active dimerization and phosphorylation of TrkB alleviate binge drinking‐induced neurodegeneration as receptor‐target artificial neurotrophin. Last, we investigated the activation of Akt and MAPK (also called ERK), two downstream effectors of TrkB.^[^ ^6^, ^29^ ^]^ The levels of activated (phosphorylated) Akt and ERK were apparently increased by bSWNTs (Figure S15). These data prove that bSWNTs have potent effects that imitate BDNF in a TrkB‐dependent manner. Collectively, the results show that bSWNTs act as an “artificial BDNF” to protect against neuronal injury and to potentially prevent EtOH‐induced NDD.

## 4 DISCUSSION

SWNTs have been extensively studied in biomedical due to their favorable characteristics, including high electrical conductivity, excellent bundle strength, and large surface areas. However, the lack of technologies to sort their structures impedes their progress in the biomedical field. We have developed new methods to purify the precise structure of SWNTs. In this study, we choose three types of SWNTs (iSWNT, bSWNT, dSWNT), and bSWNT is the most effective fraction for the therapy of binged drinking‐induced NDD according to the behavioral tests. These efforts allow us to establish the structure‐function relationship, and observe maximal therapeutic effects with low dosage administration (40 ng/rat).

In this study, SWNTs per se are no longer used as simple drug vehicles but serve as active therapeutic components. As artificial neurotrophins, our study emphasizes that bSWNTs display the most promising potentials for Trk receptors with high affinity and possess a much longer half‐life than BDNF (<10 min).^[^ ^30^ ^]^ Single microinjection of bSWNTs is efficacious for at least 15 days. These novel “artificial neurotrophins” are urgently to expand to other NDD therapy, because many studies reported the benefits of neurotrophic therapy against a variety of human diseases such as ALS, AD, PD, autism, schizophrenia, and alcohol exposure.^[^ ^31^ ^]^ On the other hand, large epidemiological studies have additionally identified binge drinking as a major risk for developing severe NDDs.^[^ ^32^ ^]^ Additionally, a series of studies also showed that bSWNTs exerted multiple neuroprotective effects in multiple disease models. Ni and colleagues observed that chemically functionalized water‐soluble SWNTs treatment could modulate neurite outgrowth by increasing the length in cultured rat neurons.^[^ ^33^ ^]^ In an in vivo study, authors found that SWNTs administration significantly protected the brains of treated rats from ischaemic injury in a transient middle cerebral artery occlusion model.^[^ ^34^ ^]^ Similarly, our previous studies also indicated that the AD‐induced dysfunction of glial cells and methamphetamine‐induced addiction behaviors could be reversed by SWNTs treatment.^[^ ^7^, ^8^ ^]^ Apart from this, evidence showed that SWNTs treatment did not influence the locomotion of zebrafish at the dosage of 100–1000 ng per animal.^[^ ^35^ ^]^ We also previously observed that i.c.v. microinjection of SWNTs (1 and 2 ng) had no effect on animal body weight, feeding, drinking, or locomotor behavior.^[^ ^8^ ^]^ In the present study, we also observed that SWNTs administration significantly reversed the alcohol‐induced neurodegeneration without influencing the locomotion as detected by the NOR tests, indicating SWNTs treatment did not influence the normal behavior of animals. Therefore, we believe that bSWNTs deserve further research as “artificial neurotrophins” for the treatment of other NDDs.

Identification of the endogenous target for nanomaterials is necessary and critical progress during nanomedicine translation to the clinic, while most of the studies pay attention to assess their functions. Besides the therapeutic effects of bSWNTs stated above, we showed for the first time that bSWNTs could interact with the TrkB receptor and further trigger its dimerization by altering its conformation and phosphorylation of tyrosine residues to support neuronal survival, growth, differentiation, and proliferation. Compared to other studies that identified drug target for small molecules, target for nanomedicine is difficult to investigate with relatively limited techniques. In the present work, we first provide experimental evidence of Trk receptor as a target for bSWNTs in the brain. A mechanistic interpretation is based on the ultimate proof for SWNT‐target validation confer comparison of functional examination, gene knockdown, determination of TrkB structural transformation, and regulation of its downstream. Collectively, bSWNTs may provide potential solutions to the daunting challenges associated with therapy for neurodegeneration, and may open up new avenues to overcome present limitations for target identification of nanomaterials.

## CONFLICT OF INTEREST

The authors declare that they have no conflict of interest.

## ETHICS STATEMENT

All experiments were conducted according to the National Institutes of Health (NIH) Guide for the Care and Use of Laboratory Animals (NIH Publications No. 80‐23, revised 1996). All applicable institutional or national guidelines for the care and use of animals were followed. All animal procedures were carried out according to the Regulations for the Administration of Affairs Concerning Experimental Animals of China and approved by the Animal Care and Use Committee of Laboratory Animal Center, Nankai University (ethics approval number: 2021‐SYDWLL‐000318).

## Supporting information

Supporting information

## Data Availability

The data that support the findings of this study are available from the corresponding author upon reasonable request.

## References

[1] N. Waszkiewicz , B. Galinska‐Skok , A. Nestsiarovich , A. Kulak‐Bejda , K. Wilczynska , K. Simonienko , M. Kwiatkowski , B. Konarzewska , Dis. Markers 2018, 2018, 5623683. 30069273 10.1155/2018/5623683 PMC6057287

[2] D. W. Zeigler , C. C. Wang , R. A. Yoast , B. D. Dickinson , M. A. McCaffree , C. B. Robinowitz , M. L. Sterling , Prev. Med. 2005, 40, 23. 15530577 10.1016/j.ypmed.2004.04.044

[3] E. Darcq , N. Morisot , K. Phamluong , V. Warnault , J. Jeanblanc , F. M. Longo , S. M. Massa , D. Ron , J. Neurosci. 2016, 36, 10116. 27683907 10.1523/JNEUROSCI.4597-14.2016 PMC5039257

[4] H. L. Haun , W. C. Griffin , M. F. Lopez , M. G. Solomon , P. J. Mulholland , J. J. Woodward , J. F. McGinty , D. Ron , H. C. Becker , Neuropharmacology 2018, 140, 35. 30056122 10.1016/j.neuropharm.2018.07.031 PMC6113096

[5] S. J. Allen , J. J. Watson , D. K. Shoemark , N. U. Barua , N. K. Patel , Pharmacol. Ther. 2013, 138, 155. 23348013 10.1016/j.pharmthera.2013.01.004

[6] S. W. Jang , X. Liu , M. Yepes , K. R. Shepherd , G. W. Miller , Y. Liu , W. D. Wilson , G. Xiao , B. Blanchi , Y. E. Sun , K. Ye , Proc. Natl. Acad. Sci. U. S. A. 2010, 107, 2687. 20133810 10.1073/pnas.0913572107 PMC2823863

[7] X. Xue , L.‐R. Wang , Y. Sato , Y. Jiang , M. Berg , D.‐S. Yang , R. A. Nixon , X.‐J. Liang , Nano. Lett. 2014, 14, 5110. 25115676 10.1021/nl501839q PMC4160261

[8] X. Xue , J.‐Y. Yang , Y. He , L.‐R. Wang , P. Liu , L.‐S. Yu , G.‐H. Bi , M.‐M. Zhu , Y.‐Y. Liu , R.‐W. Xiang , X.‐T. Yang , X.‐Y. Fan , X.‐M. Wang , J. Qi , H.‐J. Zhang , T. Wei , W. Cui , G.‐L. Ge , Z.‐X. Xi , C.‐F. Wu , X.‐J. Liang , Nat. Nanotechnol. 2016, 11, 613. 26974957 10.1038/nnano.2016.23 PMC5535299

[9] L.‐R. Wang , X. Xue , X.‐M. Hu , M.‐Y. Wei , C.‐Q. Zhang , G.‐L. Ge , X.‐J. Liang , Small 2014, 10, 2859. 24677813 10.1002/smll.201303342

[10] G. Cellot , E. Cilia , S. Cipollone , V. Rancic , A. Sucapane , S. Giordani , L. Gambazzi , H. Markram , M. Grandolfo , D. Scaini , F. Gelain , L. Casalis , M. Prato , M. Giugliano , L. Ballerini , Nat. Nanotechnol. 2009, 4, 126. 19197316 10.1038/nnano.2008.374

[11] G. Cellot , F. M. Toma , Z. K. Varley , J. Laishram , A. Villari , M. Quintana , S. Cipollone , M. Prato , L. Ballerini , J. Neurosci. 2011, 31, 12945. 21900573 10.1523/JNEUROSCI.1332-11.2011 PMC6623399

[12] P. Jaatinen , J. Riikonen , P. Riihioja , O. Kajander , A. Hervonen , Alcohol 2003, 29, 91. 12782250 10.1016/s0741-8329(03)00002-8

[13] J. A. Obernier , T. W. Bouldin , F. T. Crews , Alcohol. Clin. Exp. Res. 2002, 26, 547. 11981132

[14] Y. N. Zhao , F. Wang , Y. X. Fan , G. F. Ping , J. Y. Yang , C. F. Wu , Behav. Brain Res. 2013, 236, 270. 22985845 10.1016/j.bbr.2012.08.052

[15] J. A. Obernier , A. M. White , H. S. Swartzwelder , F. T. Crews , Pharmacol. Biochem. Behav. 2002, 72, 521. 12175448 10.1016/s0091-3057(02)00715-3

[16] T. Zal , N. R. Gascoigne , Biophys. J. 2004, 86, 3923. 15189889 10.1529/biophysj.103.022087 PMC1304294

[17] L. Wang , L. Zhang , X. Xue , G. Ge , X. Liang , Nanoscale 2012, 4, 3983. 22628008 10.1039/c2nr30346a

[18] J. Y. Yang , X. Xue , H. Tian , X. X. Wang , Y. X. Dong , F. Wang , Y. N. Zhao , X. C. Yao , W. Cui , C. F. Wu , Pharmacol. Ther. 2014, 144, 321. 25017304 10.1016/j.pharmthera.2014.07.002

[19] K. Nixon , Hippocampus 2006, 16, 287. 16421863 10.1002/hipo.20162

[20] A. Cippitelli , M. Zook , L. Bell , R. Damadzic , R. L. Eskay , M. Schwandt , M. Heilig , Neurobiol. Learn. Mem. 2010, 94, 538. 20849966 10.1016/j.nlm.2010.09.006 PMC2975859

[21] D. H. Lee , J. Y. Jeong , Y. S. Kim , J. S. Kim , Y. W. Cho , G. S. Roh , H. J. Kim , S. S. Kang , G. J. Cho , W. S. Choi , Anat. Cell Biol. 2010, 43, 194. 21212859 10.5115/acb.2010.43.3.194 PMC3015037

[22] W. T. Su , Y. A. Shih , Biomed. Mater. Eng. 2015, 26, S189. 26405961 10.3233/BME-151305

[23] X. Li , D. Sun , X. Li , D. Zhu , Z. Jia , J. Jiao , K. Wang , D. Kong , X. Zhao , L. Xu , Q. Zhao , D. Chen , X. Feng , Biomater. Sci. 2017, 5, 849–859. 28294240 10.1039/c7bm00068e

[24] J. Du , L. Feng , E. Zaitsev , H. S. Je , X. W. Liu , B. Lu , J. Cell Biol. 2003, 163, 385. 14581459 10.1083/jcb.200305134 PMC2173520

[25] K. Namekata , H. Watanabe , X. Guo , D. Kittaka , K. Kawamura , A. Kimura , C. Harada , T. Harada , Genes Cells 2012, 17, 688. 22734669 10.1111/j.1365-2443.2012.01616.x

[26] P. Tapley , F. Lamballe , M. Barbacid , Oncogene 1992, 7, 371. 1312698

[27] P. Casalbore , I. Barone , A. Felsani , I. D'Agnano , F. Michetti , G. Maira , C. Cenciarelli , J. Cell. Physiol. 2010, 224, 710. 20432466 10.1002/jcp.22170

[28] G. Leal , D. Comprido , C. B. Duarte , Neuropharmacology 2014, 76, 639. 23602987 10.1016/j.neuropharm.2013.04.005

[29] C. F. Ibanez , L. L. Ilag , J. Murray‐Rust , H. Persson , EMBO J. 1993, 12, 2281. 8508763 10.1002/j.1460-2075.1993.tb05882.x PMC413458

[30] M. Wurzelmann , J. Romeika , D. Sun , Neural. Regen. Res. 2017, 12, 7. 28250730 10.4103/1673-5374.198964 PMC5319242

[31] X. Du , R. A. Hill , Neurochem. Int. 2015, 89, 170. 26220903 10.1016/j.neuint.2015.07.021

[32] R. C. Gardner , K. Yaffe , Mol. Cell. Neurosci. 2015, 66, 75. 25748121 10.1016/j.mcn.2015.03.001 PMC4461453

[33] Y. Ni , H. Hu , E. B. Malarkey , B. Zhao , V. Montana , R. C. Haddon , V. Parpura , J. Nanosci. Nanotechnol. 2005, 5, 1707. 16245532 10.1166/jnn.2005.189

[34] H. J. Lee , J. Park , O. J. Yoon , H. W. Kim , D. Y. Lee , D. H. Kim , W. B. Lee , N. E. Lee , J. V. Bonventre , S. S. Kim , Nat. Nanotechnol. 2011, 6, 121. 21278749 10.1038/nnano.2010.281 PMC4113082

[35] G. E. Weber , L. Dal Bosco , C. O. Goncalves , A. P. Santos , C. Fantini , C. A. Furtado , G. M. Parfitt , C. Peixoto , L. A. Romano , B. S. Vaz , D. M. Barros , Toxicol. Appl. Pharmacol. 2014, 280, 484. 25168427 10.1016/j.taap.2014.08.018

