# Supplementary material for: Receptor‐targeting nanomaterials alleviate binge drinking‐induced neurodegeneration as artificial neurotrophins
Source: Exploration (Beijing). 2021 Sep 1;1(1):61–74. doi: 10.1002/EXP.20210004 (PMC10291571; doi:10.1002/EXP.20210004)
Supplement: Supplementary file 1 — Supporting information [file EXP2-1-61-s001.docx]

**Receptor-Targeting Nanomaterials Alleviate Binge Drinking-induced Neurodegeneration as Artificial Neurotrophins**

*Jingyu Yang, Lirong Wang,* *Liwen Huang, Xiaohang Che, Zhen Zhang, Chunxiao Wang, Lihuan Bai, Ping Liu, Yanan Zhao, Xiaomei Hu, Bingyang Shi, Yuequan Shen, Xing-Jie Liang, Chunfu Wu*, Xue Xue**

J. Yang, X. Che, Z. Zhang, P. Liu, Y. Zhao, X. Hu, C. Wu

Department of Pharmacology, Shenyang Pharmaceutical University, Shenyang, P. R. China

L. Huang, C. Wang, L. Bai, Y. Shen, X. Xue

State Key Laboratory of Medicinal Chemical Biology, College of Pharmacy, Nankai University, Tianjin, P. R. China

L. Wang

CAS Key Laboratory of Standardization and Measurement for Nanotechnology, National Center for Nanoscience and Technology of China, Beijing, P. R. China

X. Hu, X.-J. Liang

CAS Center for Excellence in Nanoscience, CAS Key Laboratory for Biomedical Effects of Nanomaterials and Nanosafety, National Center for Nanoscience and Technology of China, Beijing, P. R. China

B. Shi

International Joint Center for Biomedical Innovation, School of Life Sciences, Henan University, Kaifeng, Henan, P. R. China

*Correspondence:

Prof. Chunfu Wu: E-mail: wucf@syphu.edu.cn

Prof. Xue Xue: E-mail: xuexue@nankai.edu.cn


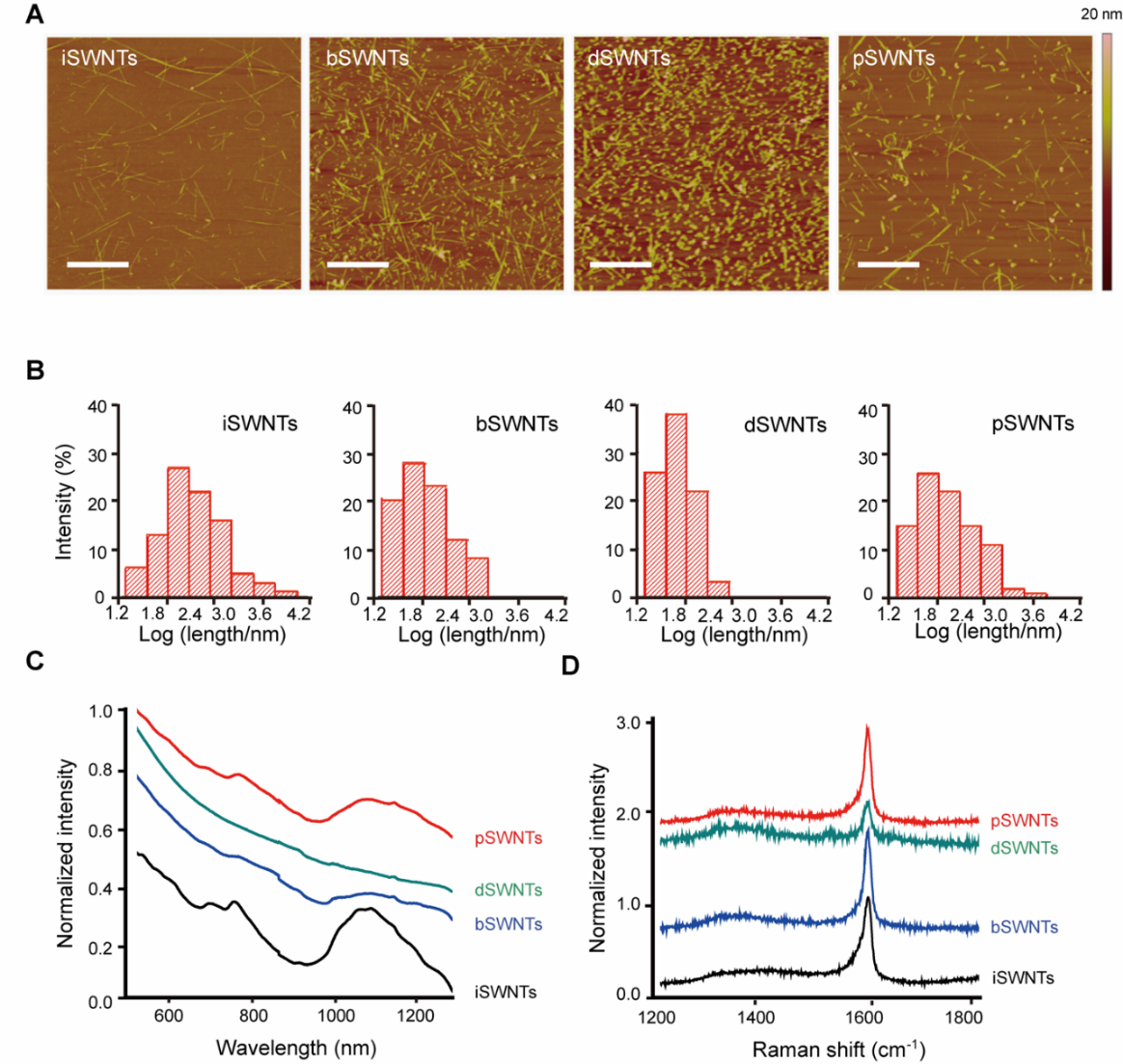


**Fig. S1**. Characterization of different types of SWNT. (A) AFM images of the four SWNT types. Scale bar; 1 µm. (B) Size distribution of the four SWNTs. (C) The visible-near-infrared (vis-NIR) absorption spectra of the four SWNTs. For the UV-vis spectrum, peaks in the 900-1,300 nm range are due to the lowest E11 sub-band absorption for semiconductors, whereas peaks in the 550-900 nm range correspond to the second E22 sub-band transitions for metals. (D) Raman spectra, with excitation at 633 nm, of the four SWNTs. The density of defects in the SWNTs is reflected by the peak ratio of D (disordered carbon signal) to G (graphitic signal) bands in the Raman spectra.


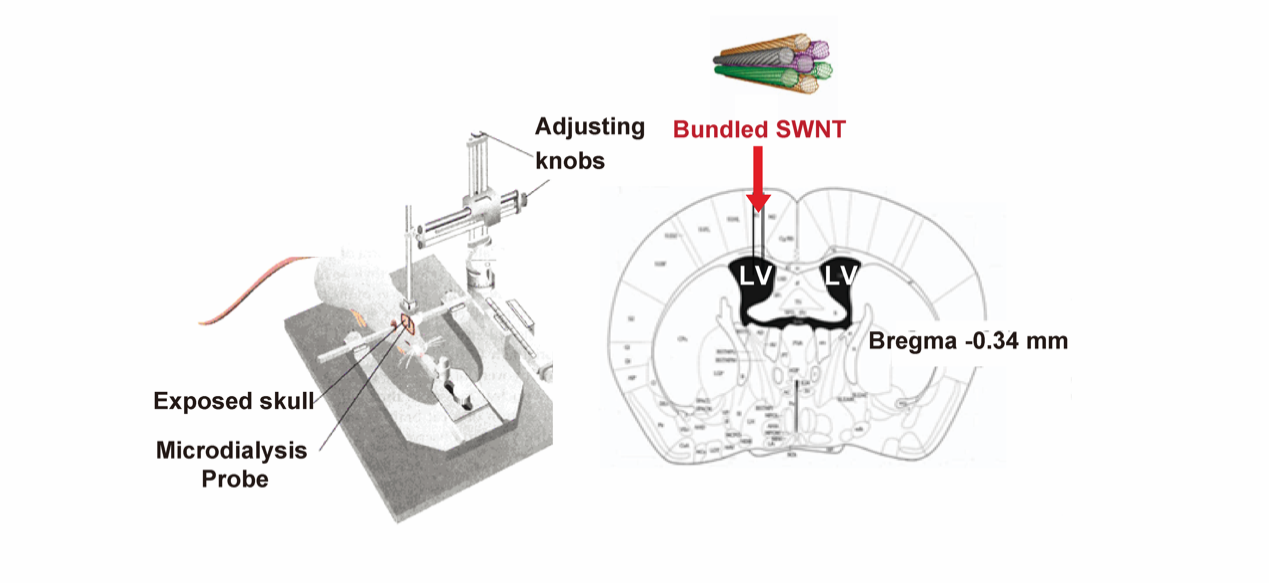


**Fig. S2**. Schematic of the procedure for intracerebroventricular injection (i.c.v.) of SWNTs in rat brain.

**
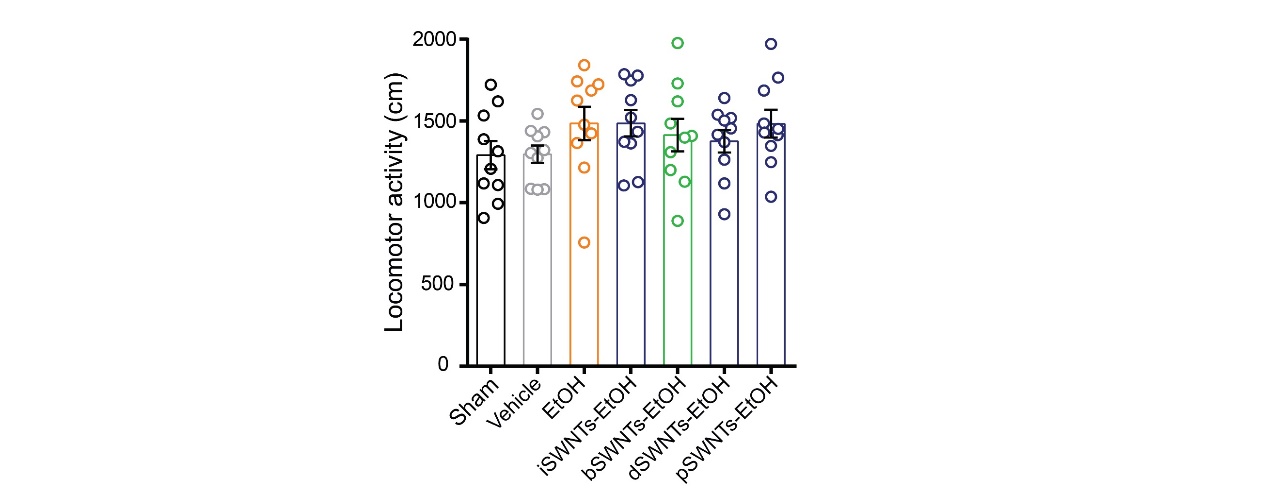
**

**Fig. S3**. Locomotor activity. No differences were observed after administration of EtOH or different types of SWNT. p > 0.05 versus EtOH group, one-way ANOVA; n=10 rats per group.

**
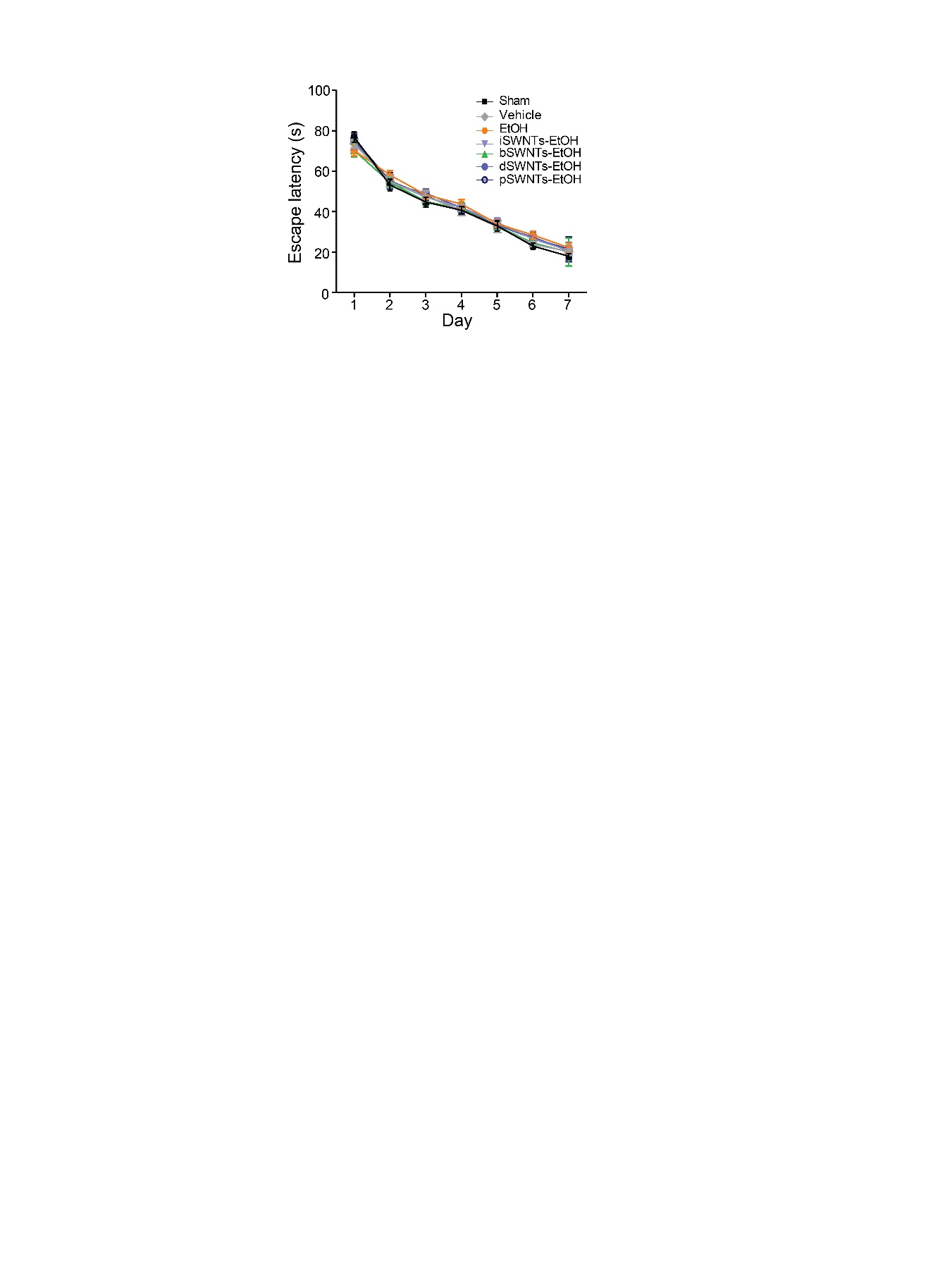
**

**Fig. S4**. Results of the Morris water maze during navigation task. No differences in the escape latency were observed during 7 days navigation task. p > 0.05 versus EtOH group, one-way ANOVA; n=8 rats per group.


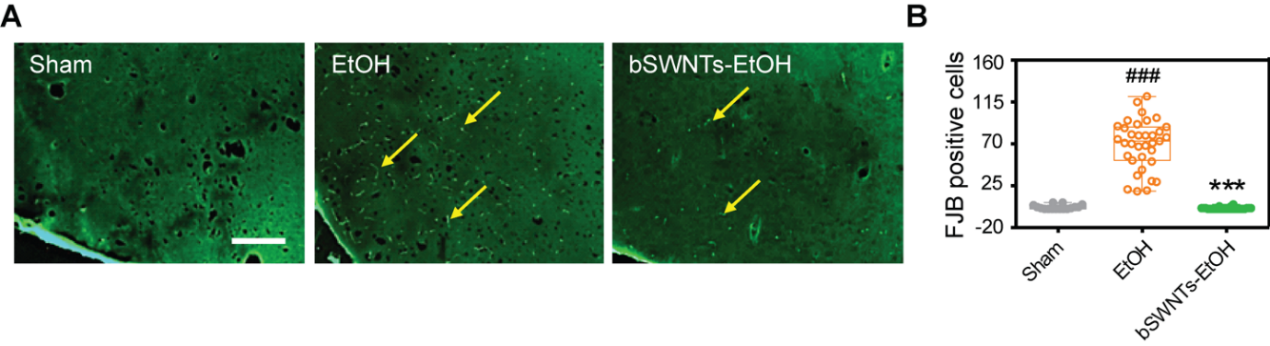


**Fig. S5**. bSWNT inhibited neuronal cell death induced by EtOH. (A) Photomicrographs showing degenerating neurons (yellow arrows), stained bright green with FJB in the prefrontal cortex region. Scale bar, 100 µm. (B) Quantitative analysis of FJB^+^ cells in the prefrontal cortex. Data are presented as mean number of FJB-positive cells/mm^2^ ± SEM. ^###^p< 0.001 versus the sham group; ***p < 0.001 versus the EtOH group; n=16 sham, n=35 EtOH, n=28 bSWNTs-EtOH from 4 rats.

**
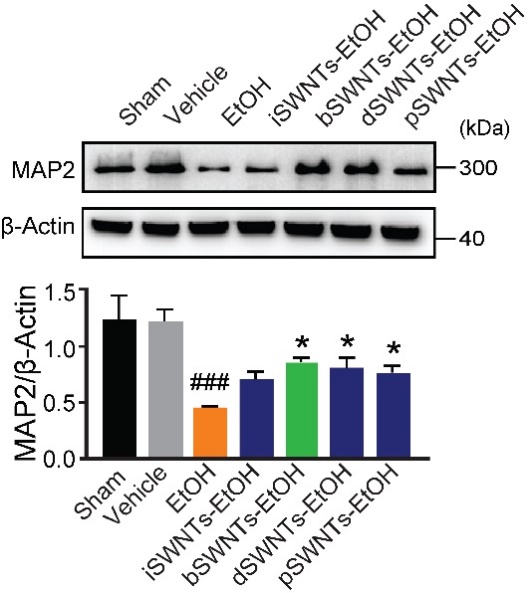
**

**Fig. S6**. MAP2 expression and quantitative analysis in hippocampus detected by western blotting. Data are presented as means ± SEM. ^###^p< 0.001 versus the sham group; *p < 0.05 versus the EtOH group; n=4 from 3 rat brains.


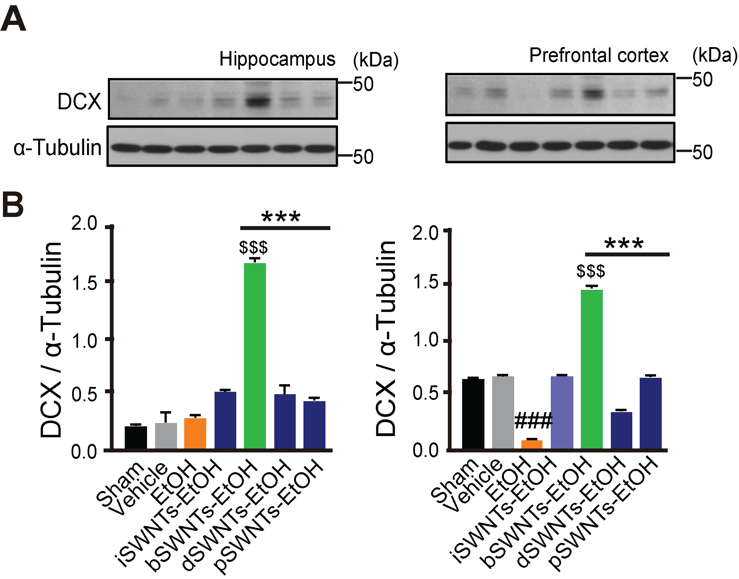


**Fig. S7**. bSWNTs promoted the expression of doublecortin (DCX). (A) Doublecortin (DCX) expression in hippocampus and prefrontal cortex detected by western blotting. Samples were obtained from the brains of 4 rats. (B) Quantification of DCX expression from the western blots in panel (A) DCX expression in hippocampus (left) and prefrontal cortex (right) brain regions was significantly higher in the bSWNT group than in the EtOH group. Data are presented as means ± SEM; ^###^p < 0.001 versus the vehicle group; ***p < 0.001 versus the EtOH group; ^$$$^p < 0.001 bSWNTs versus other SWNT treatment groups.

**
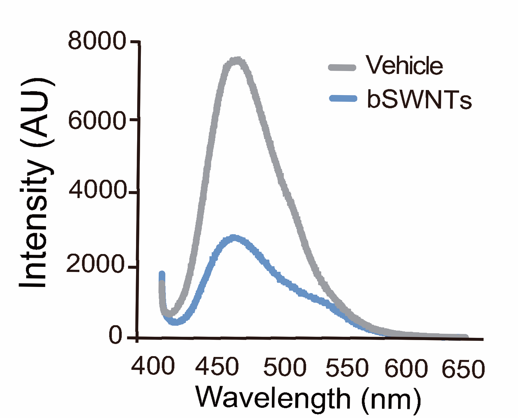
**

**Fig. S8.** bSWNT was added to cell lysate expressing GFP-TrkB or a blank control, and the fluorescence intensity was detected by a fluorescence spectrophotometer. bSWNTs quenched the GFP fluorescence via binding to GFP-TrkB.


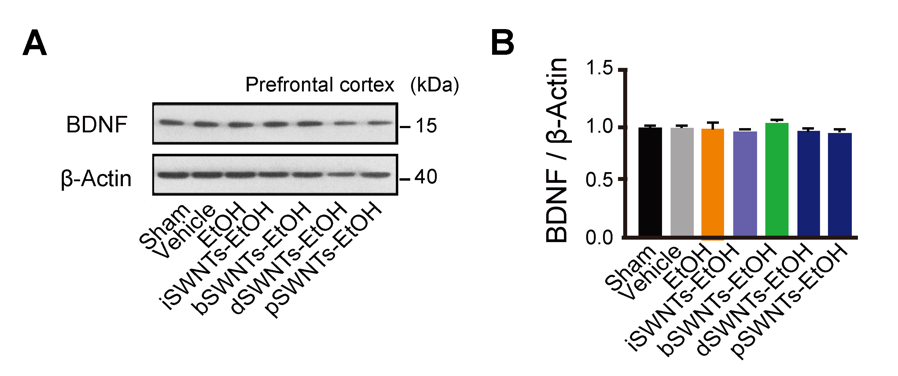


**Fig. S9.** Different types of SWNTs do not affect the expression of BDNF. (A) Immunoblots and quantitative results (B) showing that the expression of BDNF in prefrontal cortex has no obvious changes were observed after different types of SWNTs treatments.


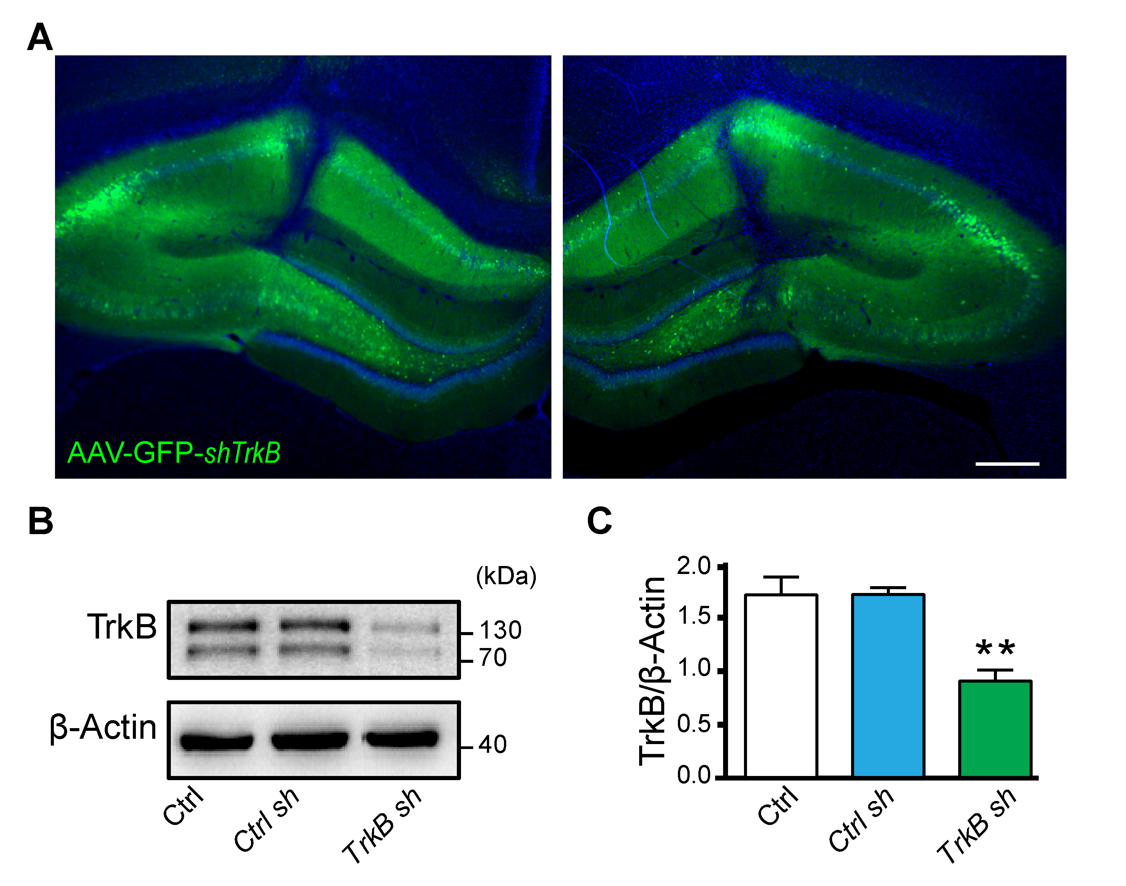


**Fig. S10**. Knockdown of TrkB in the dentate gyrus (DG) region of hippocampus in rats. (A) Photomicrographs showing the injection of GFP-*shTrkB*. Scale bar; 200 µm. (B) Immunoblots and quantitative results (C) showing the expression of TrkB after TrkB knockdown. **p < 0.01 versus *Ctrl sh* group; n=3 rats per group.

**
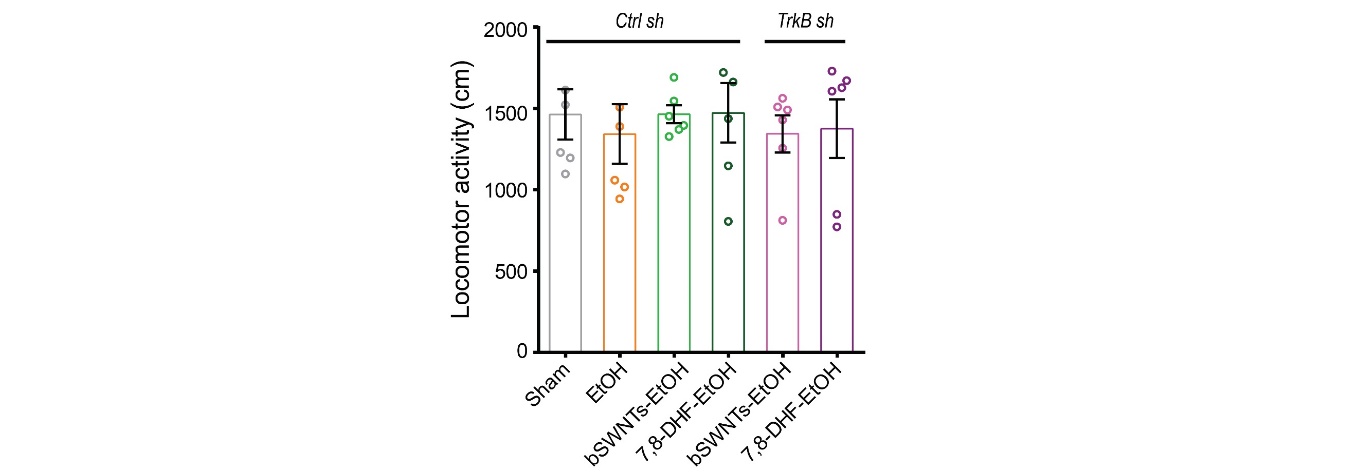
**

**Fig. S11**. Locomotor activity is not affected by bSWNTs, 7,8-DHF administration or TrkB knockdown. p > 0.05 versus EtOH group, one-way ANOVA; n=6 rats per group.

**
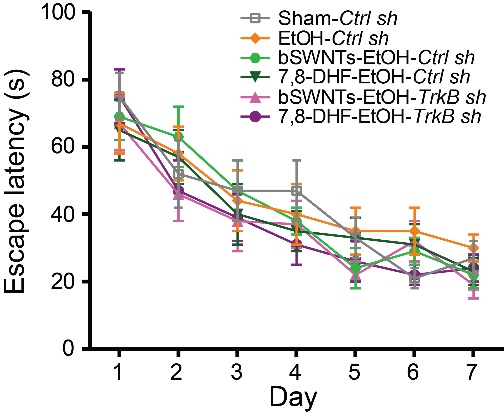
**

**Fig. S12**. Results from the Morris water maze during navigation task. Neither bSWNTs nor TrkB knockdown caused any difference in escape latency during 7 days navigation task. p > 0.05 versus EtOH group, one-way ANOVA; n=8 rats per group.

**
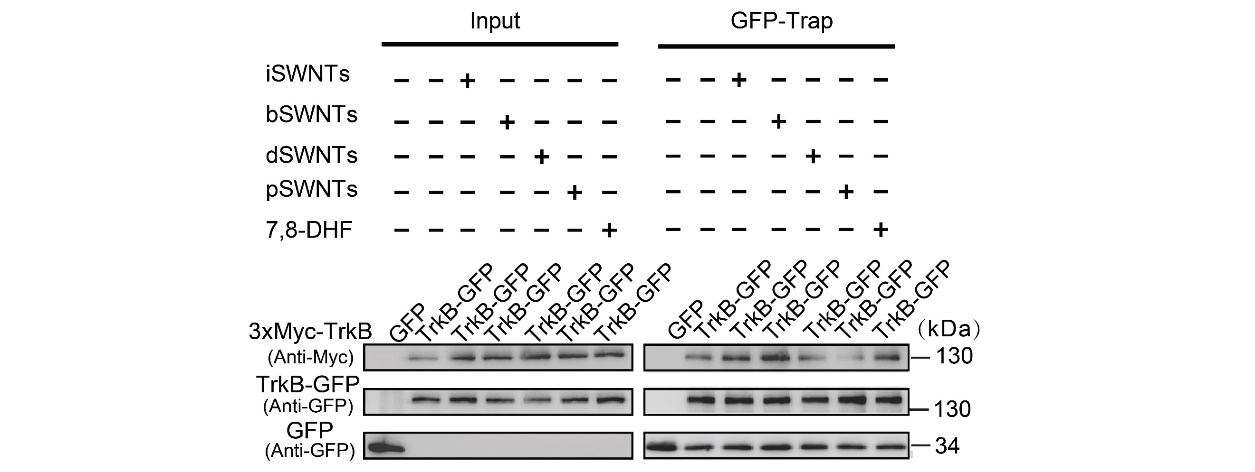
**

**Fig. S13**. In the GFP-Trap assay, Myc-TrkB was precipitated by TrkB-GFP in HEK293 cells. bSWNTs promoted TrkB dimerization more effectively than iSWNTs, dSWNTs or pSWNTs.

**
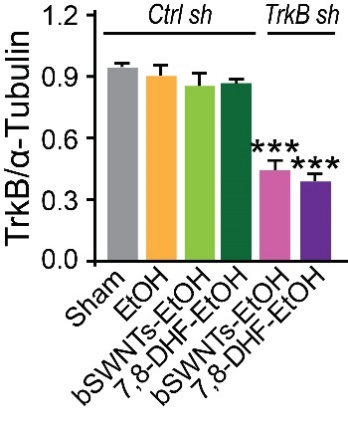
**

**Fig. S14**. Quantitative results showing the effect of TrkB knockdown on the level of TrkB protein. The expression of TrkB was dramatically decreased by TrkB knockdown. Data are presented as means ± SEM. ***p < 0.001 versus the EtOH group; n=4 rats per group.


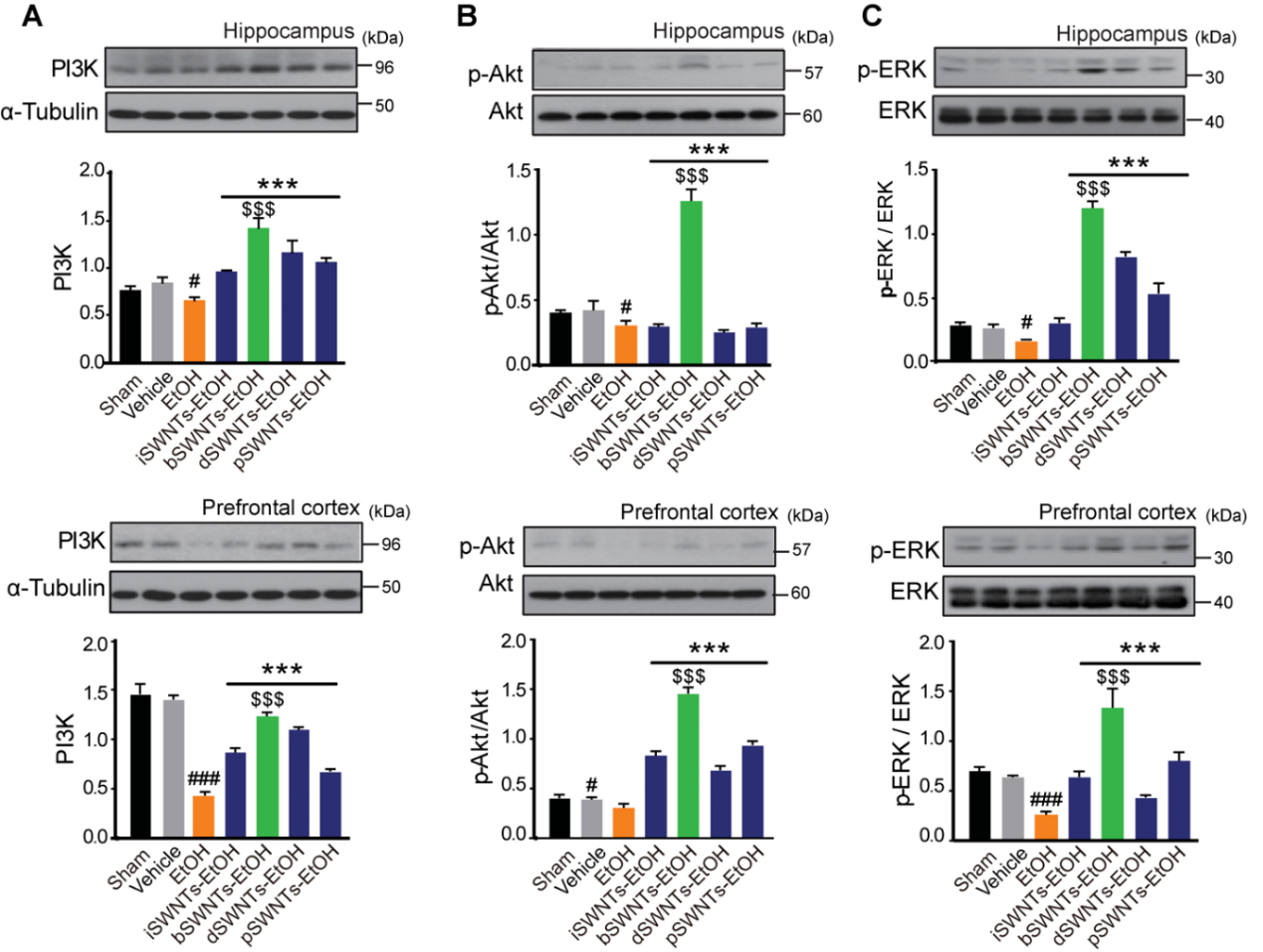


**Fig. S15**. Quantitative analysis of TrkB downstream effectors. bSWNTs activate TrkB downstream signaling pathways, as detected by western blotting. Phosphorylation of Akt and ERK are stimulated by bSWNTs in both hippocampal and prefrontal cortex regions. (A), Quantification of PI3K expression in hippocampus and prefrontal cortex after administration of different SWNTs. (B,C), The ratio of phospho-Akt and total Akt (B) and phospho-ERK and total ERK (C) was analysed by Image J. Data are presented as means ± SEM. ^#^p < 0.05, ^###^p < 0.001 versus vehicle group; ***p < 0.001 versus EtOH group; ^$$$^p < 0.001 versus other SWNT-treated groups; n=4 rats per group.
